# Engineering Slow‐Carrier Interfacial Recombination Enables Tailored Spectral Response

**DOI:** 10.1002/advs.76887

**Published:** 2026-07-31

**Authors:** Yibo Zhang, Haozhe Wang, Zeke Liu, Yun Zhong, Zheng‐Hong Lu, Nazir P. Kherani

**Affiliations:** ^1^ The Edward S. Rogers Sr. Department of Electrical and Computer Engineering University of Toronto Toronto Ontario Canada; ^2^ Department of Materials Science and Engineering University of Toronto Toronto Ontario Canada

**Keywords:** photocarrier recombination, semiconductor surfaces, slow carriers, space‐charge region, tailored spectral response

## Abstract

Photocarrier surface recombination is a critical process in optoelectronics. Here, we report that when photocarriers traverse generic semiconductor surfaces or certain carrier‐transport layers, slower carriers—arising from weaker electric‐field‐driven drift—are more likely to be trapped by defect states and subsequently recombine. This process can be engineered to unlock emergent optoelectronic functionalities. Proof‐of‐concept experiments are proposed in which a wide space‐charge region (tens of µm in width) is created in a semiconductor, and photocarriers are generated at locations with distinct electric potentials to observe their surface collection rates. The transport of photocarriers is intentionally impeded to distinguish their dynamics. We examine a variety of interfaces, including direct electrical contacts, surfaces with intentionally introduced defects, and defective organic contacts. It is observed that carriers generated in regions of low electric potential preferentially recombine, offering the possibility for tailored spectral response in semiconductor heterointerfaces. By manipulating slow‐carrier recombination, a silicon (Si) narrowband photodetector with ∼100 nm full width at half maximum (FWHM) is demonstrated. Semiconductor surfaces/interfaces are natural filters that capture weak‐drift slow carriers.

## Graphical abstract

This work demonstrates that weak‐drift slow carriers are preferentially captured at generic semiconductor surfaces/interfaces and recombine. This process is manipulated to achieve tailored spectral response. A tens of µm‐wide depletion region is created to observe surface collection rates for photocarriers generated at different positions. Various interfaces are systematically investigated, and a Si narrowband photodetector is further demonstrated.

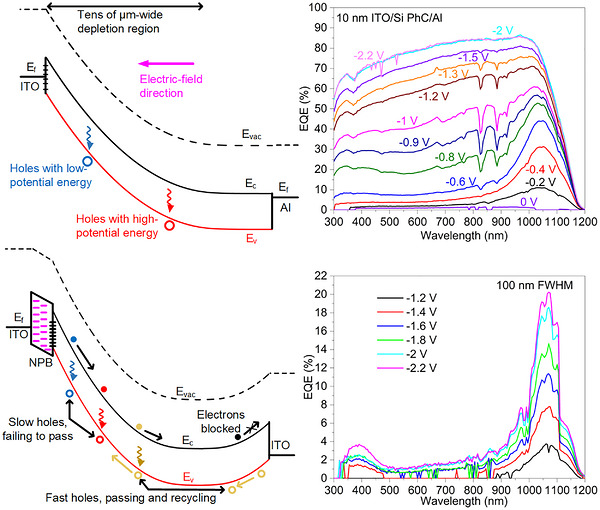

## 1 Introduction

Photocarrier recombination plays an essential role in numerous optoelectronic applications, including solar cells [1, 2, 3, 4, 5, 6, 7, 8], photodetectors [9, 10, 11, 12, 13, 14, 15, 16, 17], light‐emitting diodes [18, 19, 20], quantum computing [21, 22, 23], and photocatalysis [24, 25, 26]. In direct bandgap semiconductors, the radiative recombination process occurs predominantly [27, 28, 29], whereas non‐radiative recombination dominates in indirect bandgap semiconductors, such as Si and germanium [29, 30]. Among these non‐radiative processes, surface recombination presents a critical challenge to overcome, particularly for mature crystal lattices with eliminated bulk defects (thus mitigating bulk recombination) and dopant‐free junctions, which avoid Auger recombination caused by high carrier concentrations [4, 31]. For example, by developing passivation technologies to minimize photocarrier surface recombination, single‐junction Si solar cells have achieved conversion efficiencies exceeding 26% [7, 8], and photodetectors have attained broadband external quantum efficiencies (EQE) of above 96% [11, 12, 13]. Accordingly, the investigation of photocarrier surface recombination has been a fundamental research topic for several decades, encompassing both experimental and theoretical studies. Noteworthy landmarks include the Shockley–Read–Hall (SRH) statistics [32, 33], experiments revealing minority carrier trapping and relaxation [34, 35, 36, 37, 38], and advances in modern optoelectronics, where researchers employ ultrafast laser spectroscopy to uncover photocarrier dynamics [39, 40, 41], and manipulate photocarriers for multifunctional devices [12, 14, 15, 42]. Current research on photocarrier surface recombination has a universal drawback: All photocarriers within a semiconductor absorber are treated as having the same probability of passing through the interface. Typically, a constant recombination velocity, usually characterized by the minority‐carrier lifetime [3, 7, 43, 44], is assumed for all surface‐approaching photocarriers to assess the interface quality. However, photocarriers are not identical with respect to surface extraction—each charge carrier in a semiconductor may have a different probability of passing through the surface for extraction. Namely, the charge carrier's surface collection rate depends not only on the surface conditions but also on the particle's “transport history” [45]. A classic example is that in Si solar cells, photocarriers generated by UV light are more sensitive to surface recombination than those generated by other wavelengths [7, 30]. To date, configuring proper optoelectronic devices to directly observe photocarrier generation and collection has been rarely established, especially for generic semiconductor surfaces and carrier‐transport dielectrics. Separately considering the dynamics of individual photocarriers in optical absorbers has not yet been extensively explored, although it offers a promising approach for advancing multifunctional optoelectronics.

In this work, we suggest that slow carriers (due to weak drift) preferentially recombine at generic semiconductor surfaces as well as within certain carrier‐transport dielectrics—a process that can be precisely engineered for tailored spectral response. For an identical optical absorber, a range of spectral responses is observed by manipulating slow‐carrier interfacial recombination. A wide depletion region (tens of µm in width) is employed within a semiconductor [10, 11, 45]. Light with different wavelengths illuminates the samples to excite photocarriers at varying positions within this depletion region, so that these photocarriers are distinguished by an electric potential gradient. Unlike conventional optoelectronic devices, which are designed to enhance carrier transport and collection, we propose experiments that hinder photocarrier transport to observe their surface collection rates. We find that photocarriers generated in the low electric potential region tend to recombine preferentially at surfaces/interfaces—explaining the origin of charge passing through surfaces driven by the electric field. A commercially available Si substrate is used to configure the devices. Various surfaces are examined. First, we directly contact a transparent 10‐nm‐thick indium tin oxide (ITO) electrode with the Si surface and observe a collection gradient for photocarriers: Those generated by short wavelengths (e.g., 300–500 nm, absorption near the surface) are more likely to be captured by the interface than those generated by long wavelengths (e.g., 900–1000 nm, absorption far from the surface). Next, a layer of silicon dioxide (SiO_2_) is sputtered onto the 10 nm ITO to intentionally introduce surface defects. It is observed that a significant proportion of short‐wavelength (e.g., 300–500 nm)‐generated photocarriers can no longer be collected for the defective interface, even under a large voltage bias. Additionally, an up‐to 40‐nm‐thick N, N′‐Di(1‐naphthyl)‐N, N′‐diphenyl‐(1,1′‐biphenyl)‐4,4′‐diamine (NPB) thin film is deposited as a defective organic hole transport layer (HTL) to intentionally block hole transport, for which a similar wavelength‐dependent photocarrier collection gradient is obtained. By only permitting fast (due to strong drift) photoholes to pass through the surface/dielectrics, an ultra‐narrow‐band Si photodetector with ∼100 nm FWHM is demonstrated, highlighting the potential of utilizing semiconductor surfaces/interfaces as natural filters to selectively fail or pass charge carriers. This tailored photoresponse holds great promise for emerging technologies, such as artificial intelligence, computational imaging, and Si‐based quantum chips.

## 2 Results and Discussion

### 2.1 Experimental Mechanisms and Device Structures

**FIGURE 1:**
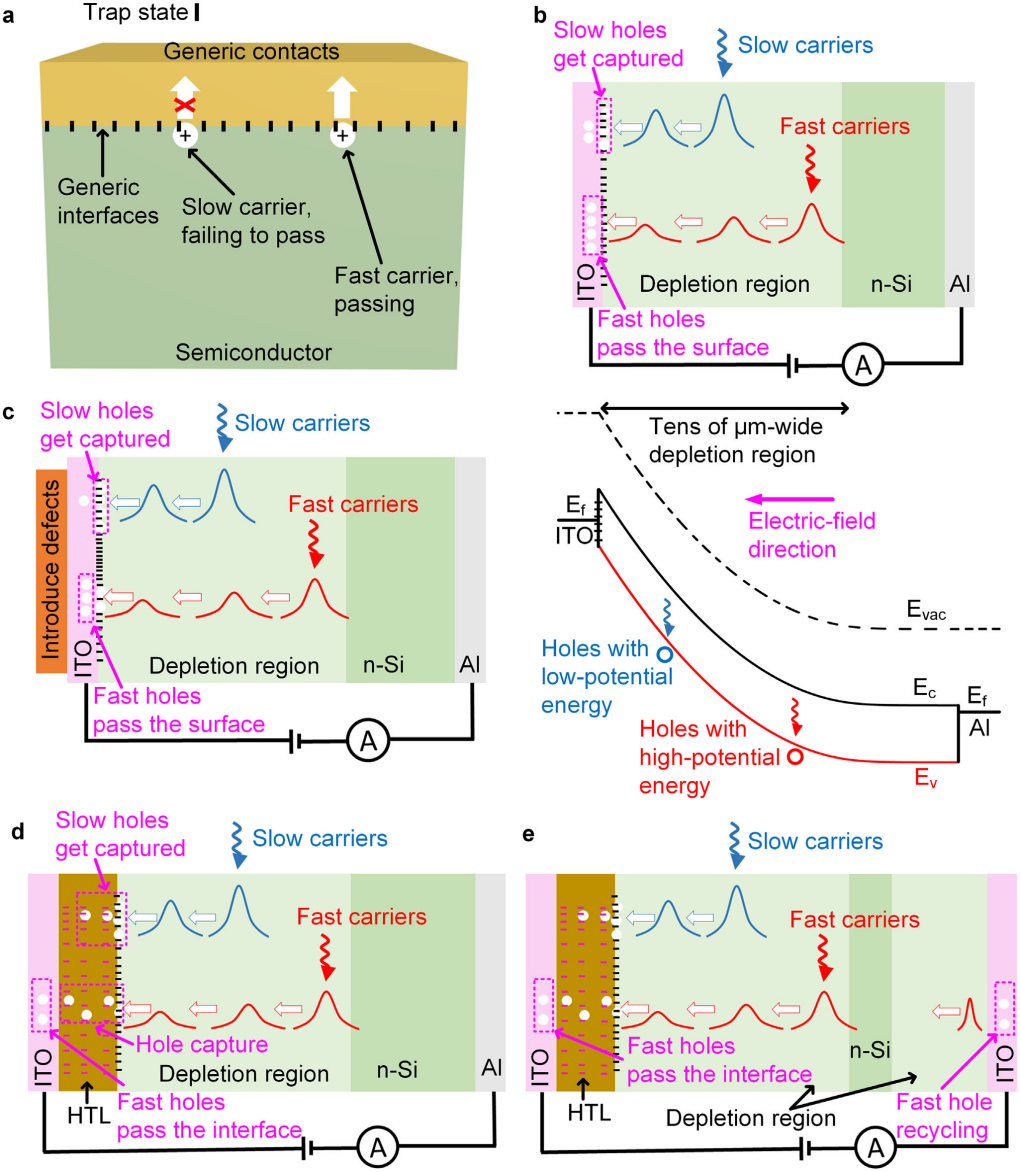
(a) Schematic illustration showing that weak‐drift slow carriers are more likely to recombine at trap states for generic semiconductor surfaces/interfaces. (b) An ITO/n‐Si device, where photocarriers are generated at different positions within n‐Si to observe their collection efficiency. A tens of µm‐wide depletion region is created within n‐Si, as indicated by the energy band diagram (Negative voltage bias is applied to ITO). Al is used as the Ohmic contact. Note that the energy band diagrams in this work are schematic and not drawn to scale. Photoholes generated in the low electric potential region within the depletion region are referred to as slow carriers, as they have weak drift dynamics when approaching the surface. In contrast, carriers generated in the high electric potential region are referred to as fast carriers. (c) Defects are intentionally introduced at the semiconductor surface to observe the carrier collection rate. (d) An organic defective HTL is used to intentionally block photoholes. Slow carriers can be trapped by both interfaces and HTL. (e) Another ITO Schottky contact (right side) is introduced to recycle fast carriers, which successfully pass through the interfaces and dielectrics from the left contact.

The experimental mechanisms and device structures are described in Figure 1. In Figure 1a, for generic semiconductors, photocarriers arriving at the interface with shorter interfacial residence times (due to stronger electric‐field‐driven drift, labelled as fast carriers) are less likely to be trapped by surface states and are therefore more likely to be extracted. Photocarriers with longer interfacial residence times (slow carriers) are more likely to be captured and fail to pass. Specifically, photocarriers generated in different regions of a semiconductor may exhibit varying transport dynamics as they approach the surface or interface, leading to different surface recombination velocities and collection probabilities. In this work, we employ a wide depletion region in semiconductors and generate photocarriers at varying positions within this region to observe their surface‐collection probability. It is demonstrated that the collection probability depends on where photocarriers are generated relative to the depletion‐region field, which sets their drift‐assisted transport and the degree of competition between trap capture/recombination and extraction. This process can be manipulated to tailor the spectral response.

First, we demonstrate this effect in Figure 1b using a direct electric‐contact device. A 10‐nm‐thick transparent ITO layer is deposited on the natural surfaces of n‐Si. This nanometer‐thin ITO forms a Schottky contact with n‐Si, inducing a wide depletion region (tens of µm wide) by selecting a low Si doping concentration (∼1.5 × 10^12^ cm^−3^). The effective Schottky barrier height at the ITO/n‐Si interface depends on the ITO work function, Si doping, and interface states [46]. A voltage bias is applied to change the electric potential of the depletion region. The detailed theoretical simulation can be found elsewhere [45]. Accordingly, for photocarriers generated at different positions within this depletion region, a potential energy gradient exists, as shown in the sketched energy band diagram (with the ITO contact negatively biased) in Figure 1b. Here, we draw the ITO/n‐Si energy band diagram in analogy to a metal–semiconductor Schottky contact, since ITO is a conductive layer with a high concentration of free electrons in its conduction band. Namely, during contact, free‐electron exchange between ITO and Si dominates the contact properties (together with surface states and the associated Fermi‐level‐pinning effect). Specifically, the free electrons in n‐Si are depleted, and accordingly, the built‐in electric field points toward the ITO contact. As such, under illumination, the photoholes (minority carriers) generated within the depletion region drift to the ITO contact, undergoing surface recombination and extraction. Bulk recombination includes band‐to‐band radiative recombination, SRH recombination, and Auger recombination. As an indirect bandgap semiconductor, Si exhibits an intrinsically negligible band‐to‐band recombination rate. Auger recombination is also suppressed by adopting dopant‐free designs and low optical injection levels. Additionally, using high‐quality float‐zone Si wafers and performing low optical injection tests minimize bulk SRH recombination. Therefore, surface/interface recombination is expected to dominate minority‐carrier loss relative to bulk recombination on the relevant transport timescales. Note that photoelectrons are the majority carriers under the small optical injection conditions (explored in this work). Their extraction relies on the quality of the Ohmic contact on the right side of n‐Si. The aluminum (Al) electrode forms a majority‐carrier Ohmic contact with n‐Si, providing sufficient electron injection capability [11]. Under our operating conditions, we do not observe significant photoconductive gain, enabling the measured EQE to be interpreted primarily in terms of minority‐carrier collection at the left (top) contact. Several factors account for the formation of the Ohmic contact and the mitigated photoconductive gain effect at the Al/n‐Si interface, including n‐Si doping, energy band alignment, and interface effects (interfacial reactions, interface states, surface dipoles, etc.). The absence of photoconductive gain effect indicates mitigated photocarrier recycling pathways, meaning photoholes can rarely be injected from the left (top) contact to the right (rear) side of the Si to complete recycling. This is explained by the large hole energy barrier from Al to n‐Si, which may arise from both energy band alignment (large band offset between Al E_f_ and Si E_v_) and interface effects. Impact‐ionization‐driven carrier multiplication is not expected under the moderate electric fields associated with the low‐doped n‐Si and the wide depletion region in these devices [45, 47]. When there is no gain, the maximum photocarrier collection rate is 100% (i.e., all generated photocarriers are collected with no loss). Given all the above factors, the minority‐carrier collection rate by the ITO contact represents the photohole surface recombination rate. In Figure 1b, we use “slow” and “fast” as relative labels for carriers generated in weaker‐electric‐potential versus stronger‐electric‐potential regions of the depletion profile, which leads to lower versus higher drift‐assisted collection probability. As holes drift through the depletion‐region field, they accelerate between scattering events; however, rapid phonon scattering leads to near‐thermal energies. Accordingly, as they approach the surface, the field‐assisted drift dynamics differ for photoholes generated in different regions, leading to distinct collection probabilities. We maintained the initial n‐Si surface without intentional chemical passivation to observe interactions between photocarriers and natural surface states. Even so, the deposition process may unavoidably cause re‐distributed surface states.

In Figure 1c, we intentionally introduce surface defects by depositing a SiO_2_ layer atop the ITO. Specifically, the nanometer‐thin ITO enables ions to diffuse into the n‐Si surface during deposition. The SiO_2_ overlayer increases absorption in Si primarily via an anti‐reflection effect, given the low optical index of SiO_2_, while sputtering can simultaneously introduce interface defects (via ion diffusion) that increase recombination. This allows for the observation of the photocarrier collection rate at a “more defective” interface. In this case, a significant proportion of the slow carriers is trapped at the surface, even under a high voltage bias, as discussed below.

In Figure 1d, a defective NPB organic HTL is deposited on the n‐Si surface, allowing hole transport to investigate the interactions between photocarriers, interfaces, and the HTL. In this case, photocarriers can be trapped by defect states at the interface and in the HTL. When the HTL thickness is increased to 40 nm, the hole‐capturing rates for both slow and fast carriers are dramatically increased, further filtering slow carriers. After replacing Al with another ITO layer in Figure 1e, the hole injection barrier from the right‐side ITO to n‐Si valence band is reduced (due to several factors, including interface effects and alignment between the ITO work function and n‐Si E_v_). This enables amplification of fast carriers. Namely, those fast carriers that succeed in passing through the left heterointerface are injected into the n‐Si from the right‐side ITO contact, facilitating photocarrier recycling and photocurrent gain effects (as demonstrated by the narrowband photodetector below).

### 2.2 Optoelectronic Characterization

**FIGURE 2:**
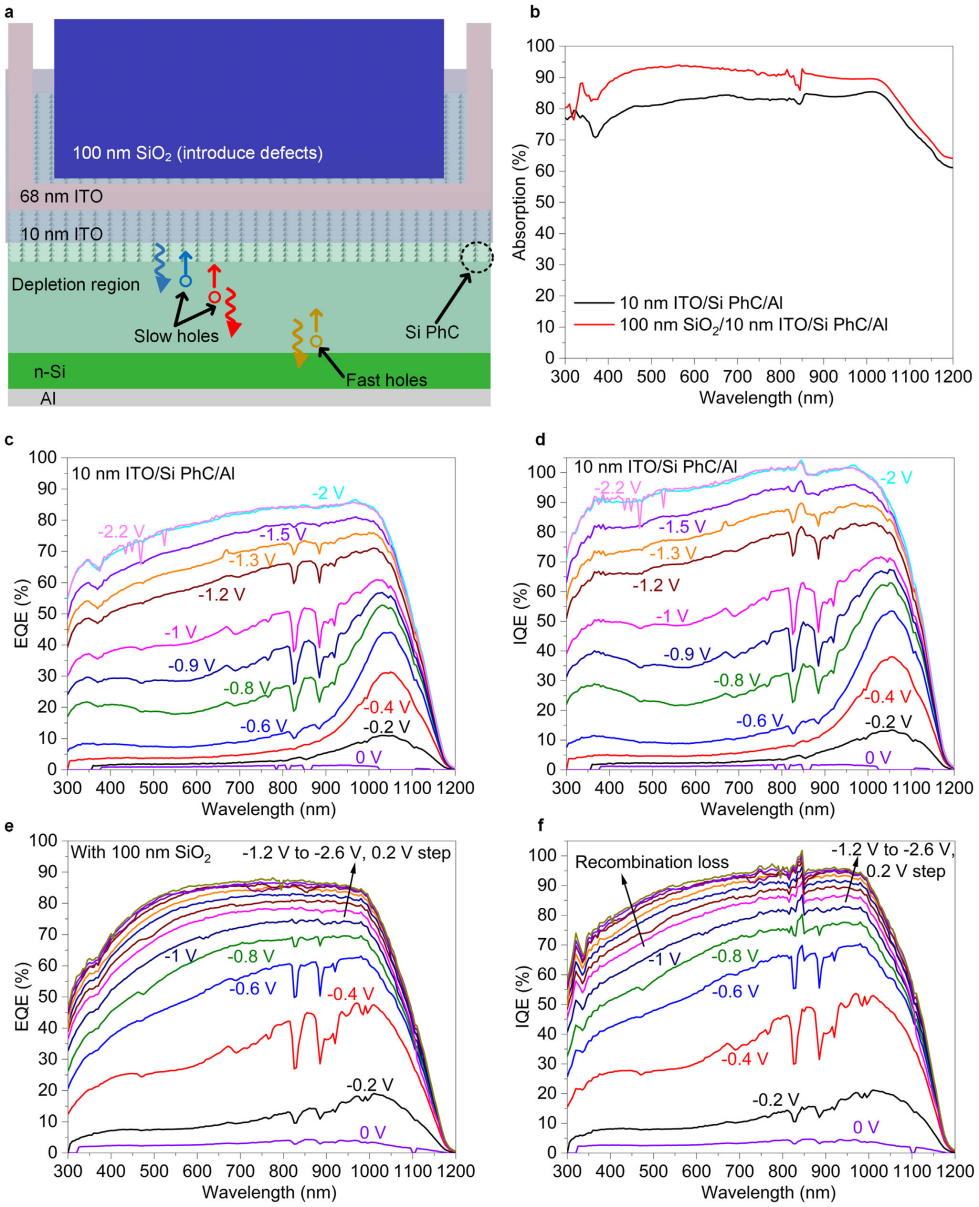
(a) Device structure, where wave‐interference photonic crystals are integrated on n‐Si. A 100‐nm‐thick SiO_2_ layer is optionally deposited on ITO to introduce defects. (b) Optical absorption rates for ITO/Si PhC/Al devices with and without 100 nm SiO_2_ on top. (c) EQE for a 10 nm ITO contact device without SiO_2_, at varying voltage biases. (d) IQE for a 10 nm ITO contact device without SiO_2_. (e) EQE for devices with 100 nm SiO_2_ to introduce defects. (f) IQE of the device with 100 nm SiO_2_.

Direct‐contact devices are demonstrated in Figure 2, corresponding to the cases in Figure 1b,c. The device structure is illustrated in Figure 2a, featuring ∼10 nm ITO as the electrical contact with n‐Si and ∼68 nm ITO as the encapsulating electrode for testing. A ∼100 nm layer of SiO_2_ is optionally deposited on top of the ∼10 nm ITO to introduce defects. Wave‐interference photonic crystals (PhCs) are integrated into Si to improve the overall absorption rate by light trapping [48, 49]. These PhCs are an array of inverse pyramids, with a nanometer‐thin mesa width between adjacent pyramids [10], as shown in Figure S1. The optical absorption is shown in Figure 2b for samples with ∼10 nm ITO/Si PhC/Al. Broadband absorption rates of ∼80% and ∼90% are obtained for devices with and without SiO_2_ on top, respectively. This ensures high optical absorption over 300–1000 nm, enabling clear observation of variation in photocarrier extraction. The enhancement in optical absorption in devices with SiO_2_ is due to the anti‐reflection effect from SiO_2_’s low refractive index. The optical absorption depth of n‐Si (Figure S2) suggests that short‐wavelength photocarriers are generated within the low‐electric‐potential region (near the surface).

The device transport properties, including dark current, effective Schottky barrier formation, interface states and Fermi‐level‐pinning effect, have been systematically investigated elsewhere [9, 10, 11, 45, 46]. By solving Poisson's equation in technology computer‐aided design (TCAD) simulations, a depletion region width of 30–40 µm is theoretically predicted under a moderate reverse bias (e.g., −1 V), for ITO/n‐Si (∼1.5 × 10^12^ cm^−3^ doping level)/Al devices [45]. This is experimentally verified in Figure S3, using capacitance–voltage (C–V) measurements. As the reverse bias increases from 1.5 to 3 V, the depletion region width increases from approximately 29 to 40 µm (Figure S3c), in good agreement with TCAD predictions. EQE results are presented in Figure 2c for devices without SiO_2_. This metric indicates the ratio of collected photocarriers to incident photons. As discussed in Figure 1b, the charge carrier collection ratio reflects the carrier loss due to surface recombination. From −0.2 to −0.6 V, a narrowband collection is obtained, where long‐wavelength photocarriers (generated far from the n‐Si surface) generally have a higher EQE than those generated at short wavelengths. The EQE peak appears at 1050–1060 nm, corresponding to the indirect bandgap edge (i.e., Si has a weak optical absorption coefficient for wavelengths above ∼1060 nm due to its indirect bandgap, which is why the EQE drops after ∼1060 nm). This narrowband photocarrier collection is accompanied by a tens of µm‐wide depletion region, where the electric potential for these photocarriers varies with wavelength. In contrast, devices with ∼1 µm‐wide depletion regions exhibit a constant EQE at 450–850 nm [9, 45], exhibiting no narrowband spectral response. This observation confirms the assumption in Figure 1b, whereby these weak‐drift photoholes are more likely to be trapped by surface states with direct electrical contacts. Note that the optical absorption of high‐energy photons (e.g., UV light) also leads to a hot‐carrier effect for these photogenerated free carriers, which is distinct from built‐in electric‐field‐driven drift. Specifically, hot carriers generated by optical absorption have no preferred momentum direction, in contrast to those moving under a preferred electric‐field direction herein. A well‐passivated hole‐collecting surface leads to nearly 100% EQE at 0 V bias with Al rear Ohmic contact [11], confirming that minority‐carrier (holes) collection represents the front‐side recombination rate as long as the Ohmic contact (Al) is sufficiently good for majority‐carrier injection. Namely, one photohole is collected by the ITO contact, while one electron is injected into the Al contact to maintain the system's electrical neutrality. In Figure 2c, as the surface potential gradually increases (under a higher applied voltage), the surface collection rate for these short‐wavelength photocarriers increases. Similarly, photocarriers at longer wavelengths exhibit higher EQE than those at shorter wavelengths over a bias range of −0.8 to −1.2 V. The EQE saturates at −2 V, reaching the maximum collection rate.

The internal quantum efficiency (IQE), defined as the ratio of collected charges to absorbed photons, is calculated in Figure 2d using the equation:

(1) IQE=EQE/Absorption

The absorption results are taken from Figure 2b. Using a 10 nm‐thin ITO layer reduces parasitic absorption from ITO. Similarly, the IQE at long wavelengths is generally higher than that at short wavelengths, confirming the recombination of these slow carriers. Notably, the IQE at 1000 nm first approaches 100% with increasing bias, whereas the IQE at 450 nm remains ∼91%. The 100% IQE confirms the limited photocurrent gain effect in the configured devices, as analyzed in Figure 1b. The approximately 9% IQE loss at 450 nm suggests that a significant portion of the photocarriers generated near the n‐Si surface cannot be fully collected due to surface recombination (thin‐ITO is transparent to 450 nm photons). Our previous work indicates that when the surface electric field is not sufficiently strong, near‐surface‐generated photocarriers cannot be fully collected in the absence of surface chemical passivation [45]. Herein, it is further concluded that the photocarrier surface collection rate is directly dependent on field‐assisted drift within the depletion region. In the absence of surface chemical passivation to maintain a low surface‐state density, those carriers that do not obtain strong drift from the field region cannot pass the interface. This claim is verified by intentionally introducing defects at the n‐Si interface, as shown in Figure 2e,f, for devices with a SiO_2_ layer atop the ITO. After the SiO_2_ layer is deposited, the introduced interface states are evidenced by the increased reverse dark current density and reduced effective Schottky barrier height (resulting from the Fermi‐level‐pinning effect, and the associated barrier‐lowering effect), confirmed in Figures S4 and S5. Similarly, in Figure 2e, the long‐wavelength EQE is generally higher than that at short wavelengths. Notably, narrowband photocarrier collection is not observed at low biases. Interestingly, despite the improved optical absorption (Figure 2b), the saturated EQE at longer wavelengths (900–1000 nm) in Figure 2e remains comparable to that in Figure 2c. This can be attributed to enhanced broadband photocarrier recombination: while the SiO_2_ layer increases overall absorption, it also introduces defects at the n‐Si surface, which, in turn, increase the overall recombination rate. As a result, the saturated EQE remains similar: 85%–86% EQE at 950 nm in both Figure 2e,c.

The IQE results in Figure 2f indicate that intentionally added surface defects influence the photocarrier recombination rate across the entire spectrum. IQE results further verify the enhanced recombination of photocarriers: With increasing bias, the long‐wavelength IQE saturates at ∼95% at 950 nm in Figure 2f, compared with that at ∼100% in Figure 2d at the same wavelength. While it is traditionally considered that photocarriers generated near the surface are strongly affected by surface recombination [30], our results further suggest that the recombination probability for photocarriers generated at different locations within the entire photoactive layer is differentially influenced as the surface‐defect density increases. The recombination loss is further analyzed by the IQE at short wavelengths: An IQE of approximately 91% is observed at 450 nm in Figure 2d (saturated at −2 V bias), which drops to about 83% in Figure 2f (−2.6 V). This indicates that a large proportion of photocarriers generated at short wavelengths fail to traverse the defective interface, even under a higher applied bias of −2.6 V.

The EQE/IQE results in the low‐bias region (−0.2 and −0.6 V) and the saturated region (−2 V) are compared for devices with and without SiO_2_ in Figure S6. Figure S6a presents the EQE results. At –0.6 V, SiO_2_ deposition leads to only a slight increase in the EQE at 1050 nm (corresponding to the narrowband response peak), whereas the broadband EQE (e.g., 400–900 nm) exhibits a much more pronounced enhancement. For instance, after SiO_2_ deposition, the EQE increases modestly from ∼44.1% to ∼51.1% at 1050 nm, but rises dramatically from ∼8.9% to ∼56.1% at 700 nm. Considering that the optical absorption enhancement introduced by SiO_2_ is modest (Figure 2b), whereas the corresponding IQE change is significantly larger (Figure S6b), the substantial broadband EQE enhancement is unlikely to originate primarily from increased optical absorption. In addition, the possibility that the low‐refractive‐index SiO_2_ layer alters the photocarrier spatial generation profile and thereby significantly influences surface carrier collection is excluded by the finite‐difference time‐domain (FDTD) simulations in Figure S7. The simulations reveal that the photogeneration profile and the carrier generation depth remain nearly unchanged after SiO_2_ deposition. Therefore, the substantial EQE variation is attributed primarily to altered device transport properties. Specifically, during SiO_2_ sputtering, ion diffusion and other unavoidable process‐induced effects, such as thermal annealing, can modify the device interfaces, thereby affecting the defect‐state distribution, carrier trapping behavior, and voltage distribution across the device and interface (Figure S4), explaining the low‐bias EQE/IQE variation at −0.6 V. At −2 V, the IQE at 700 nm decreases from ∼98.0% to ∼92.8% after SiO_2_ deposition (Figure S6b), indicating enhanced surface recombination and further confirming the introduction of additional interface defects by SiO_2_ deposition.

Note that the proposed slow‐carrier recombination effect in this work is conceptionally distinct from the interface‐trap‐assisted transport or barrier‐lowering effects. After SiO_2_ deposition, the enhanced photocarrier recombination (Figure S6b, −2 V bias) and the reduced effective Schottky barrier height (Figure S4) are due to the interface‐trap‐assisted transport and barrier‐lowering effects. In contrast, the observed wavelength‐dependent EQE/IQE gradient arises from the slow‐carrier recombination effect.

**FIGURE 3:**
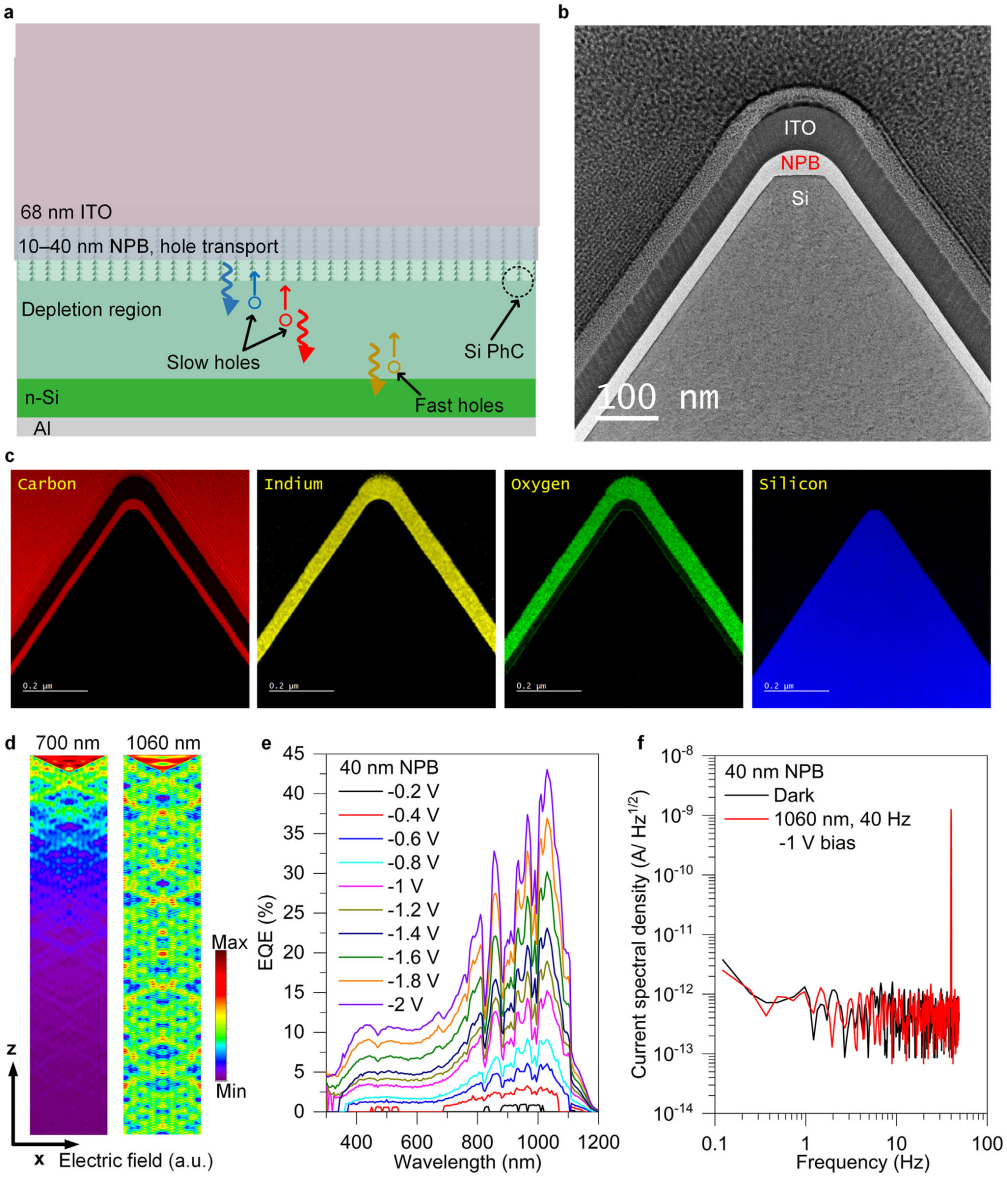
(a) Device structure, where NPB thin films with 10–40 nm thicknesses are contacting n‐Si. (b) HRTEM image showing the interface profile for the ITO/NPB/Si PhC. (c) EDS mapping results showing the carbon, indium, oxygen, and Si distribution. An ultra‐thin oxygen layer reveals the existence of native SiO_x_. (d) Electric field distribution under 700 and 1060 nm illumination, simulated by FDTD. The simulated area is 2 µm × 29.3 µm. Note: The *x* and *z* axes are not plotted to scale. (e) EQE for devices with 40 nm NPB. (f) CSD for a 40 nm NPB device with/without 1060 nm laser illumination.

Slow carriers preferentially captured by trap states are also observed in certain dielectrics, as illustrated in Figure 3. Here, we thermally evaporated an organic layer (NPB) of varying thickness onto the Si PhCs to observe the photocarrier extraction rate (corresponding to the case in Figure 1d). The device structure is depicted in Figure 3a, where a ∼68‐nm‐thick ITO layer serves as the transparent electrode, atop 10–40 nm NPB HTLs. The large energy band offset between NPB HOMO/LUMO (highest occupied molecular orbital/lowest unoccupied molecular orbital) and Si E_v_/E_c_ induces a surface depletion region on the Si side. In addition to the energy band offset, the properties of generic carrier‐transport layers are influenced by other factors such as interface passivation, interband states, and traps. In this configuration, photoholes can be trapped by defects at the n‐Si surface, within the NPB dielectric, and at the NPB/ITO interface. Conventional HTLs provide surface passivation and typically have a moderate thickness (5–15 nm) to balance surface barrier formation and efficient hole transport [4], thereby enabling high device performance. In contrast, in this work we intentionally increase the HTL thickness to 40 nm to introduce a substantial barrier to photocarriers, thereby effectively distinguishing their ability to pass through the interfaces. The introduced defects by NPB thin films are confirmed by C–V measurements at varying frequencies in Figure S8. At low frequencies (10–50 kHz), both broad and narrow capacitance peaks are observed, arising from defect levels [50]. For example, at 40 kHz, peaks appear at −0.35 and 0.09 V, and at 50 kHz, a peak appears at 0.12 V, indicating the introduced defects occupy varying energy levels with distinct response speeds. These peaks disappear at high frequencies (80 and 100 kHz) because the slow defect‐level response cannot keep pace with the fast input signal. Note that the identified defects from C–V curves may originate from NPB thin films, the NPB–Si interface, and the ITO–NPB interface.

The device interface is characterized using high‐resolution transmission electron microscopy (HRTEM), as shown in Figure 3b. HRTEM reveals ∼40 nm NPB, ∼68 nm ITO, and <100 nm mesa width for the Si PhC [49]. Energy‐dispersive X‐ray spectroscopy (EDS) mapping results show the elemental distribution of carbon, indium, oxygen, and Si (Figure 3c). The existence of a thin oxygen layer between carbon and Si indicates the presence of a native SiO_x_ layer, which is usually grown on the natural n‐Si surfaces.

Figure 3d presents the simulated electric field under illumination (700 and 1060 nm wavelengths) by FDTD simulation. Note that the simulated electric field here is from the illumination, not including that from the semiconductor's built‐in electric field. Photocarriers generated under 700 nm light are predominantly confined within the top 3 µm of the surface. In comparison, those generated by 1060 nm light are distributed throughout the entire simulation region (29.3 µm in length). It is worth mentioning that the electric‐field and photogeneration distributions (Figure S9) at 1060 nm are expected to extend beyond the simulated area used in this work, given the Si's high absorption depth at 1060 nm (Figure S2). These results support the observed variation in photocarrier collection and reinforce the assumption that photocarriers generated in low‐potential‐energy regions are more likely to recombine at defect states located at surfaces/interfaces and within certain dielectrics.

The EQE results for devices with 40 nm NPB are shown in Figure 3e. Increasing the carrier transport distance by using a ∼40‐nm‐thick NPB layer significantly raises the barrier for photoholes to reach the ITO electrode for extraction (Figure 1d), leading to an EQE gradient spanning 300–1060 nm. This trend is consistent with the results shown in Figure 2c,e, supporting the conclusion that certain dielectrics can trap charge carriers similarly to general semiconductor surfaces. The EQE characteristics of devices with 10 and 20 nm NPB layers are presented in Figure S10. No obvious wavelength‐dependent EQE gradient is observed, indicating that the spectral response is strongly thickness‐dependent. The absence of the EQE gradient can be explained by the balance between the hole‐transport distance and the surface hole‐extraction barrier. The NPB layer enhances the surface hole‐extraction barrier by several factors, such as interface passivation and surface‐field effect arising from the energy‐band offset. Specifically, rather than hindering hole transport, thin NPB layers (10 and 20 nm) enhance hole transport and extraction, thereby suppressing the EQE gradient. This interpretation is supported by comparing the 0 V bias EQE between ITO/Si PhC/Al and ITO/NPB/Si PhC/Al devices, as shown in Figure S11. For thin NPB layers, the combination of an enhanced surface hole‐extraction barrier and a proper HTL thickness enables efficient hole transport and extraction, resulting in the highest 0 V EQE for the 10 nm NPB device (Figure S11) and a non‐observed EQE gradient (Figure S10). A similar phenomenon was reported in devices employing a SiO_x_ interfacial chemical passivation layer and an ITO extraction layer, where a nearly constant broadband EQE was observed despite the presence of a wide‐depletion region [11]. In contrast, increasing the NPB thickness to 40 nm significantly hinders hole transport, thereby leading to the EQE gradient. These findings further suggest that photocarriers are not identical in terms of their recombination and extraction behaviors when transporting within certain dielectrics, paving the way for manipulating photocarriers via contact engineering in next‐generation optoelectronics. Overall, the central aim of this study is to introduce a high barrier to photocarriers and evaluate their ability to overcome it, thereby clearly observing the effects of the surface or transport dielectrics on photocarriers generated at different positions.

A molybdenum trioxide (MoO_3_) layer is employed to replace NPB as a control device, and the corresponding results are presented in Figures S12–S14. Note that MoO_3_ is commonly used as a hole‐extraction layer rather than a conventional hole‐transport layer such as NPB. Unlike conventional MoO_3_ layers, which typically employ 5–15 nm thickness for efficient hole extraction and surface barrier enhancement [31], a thicker 25 nm MoO_3_ layer is adopted here to intentionally increase the hole‐transport resistance. The 25‐nm MoO_3_ devices exhibit a higher hole‐transport resistance than that of the NPB devices, as evidenced by the current–voltage curves in Figure S12. Specifically, the forward current of the NPB devices is significantly higher than that of the MoO_3_ devices, indicating a lower hole‐transport resistance for the NPB devices. The EQE results for MoO_3_ devices are given in Figure S13. No obvious wavelength‐dependent EQE gradient is observed, suggesting that merely increasing the hole‐transport resistance or modifying the surface extraction barrier does not necessarily induce the EQE gradient. Therefore, the EQE gradient emerges only in specific carrier‐transport dielectrics and, for devices investigated in this work, mainly originates from trap‐assisted slow‐carrier recombination within the NPB thin films and their interfaces. This also agrees with the C–V and EQE results. Depositing NPB thin films introduces defects (Figure S8). A 10 nm NPB layer enhances carrier collection by balancing transport distance and surface barrier enhancement (Figure S11), whereas a 40 nm NPB layer impedes hole transport via defect‐assisted recombination (Figure 3e). Additionally, the possibility that the 25‐nm MoO_3_ has a better hole‐transport capability than that of the 40‐nm NPB, thereby accounting for the absence of the EQE gradient, can also be excluded by comparing the EQE results in Figure S14. Although the 25‐nm MoO_3_ devices have high broadband optical absorption (Figure S14a), their EQE remains below 10% even under a high bias of −3 V (Figure S14b). By comparison, the EQE of the NPB devices is substantially higher than that of the MoO_3_ devices at both −2 and −3 V, demonstrating that the 40‐nm NPB layer provides superior hole‐transport capability to the 25‐nm MoO_3_ layer (deposited with 5% O_2_ flow during sputtering). Meanwhile, it should be noted that hole‐transport resistance and hole recombination are inherently related in semiconductor devices. Therefore, it is not physically appropriate to entirely exclude the possibility that increased hole‐transport resistance also contributes to the observed EQE gradient. For example, the enhanced hole‐transport resistance may prolong the hole transit time, thereby increasing the probability of hole trapping and recombination. Nevertheless, from a more general perspective, other contributing factors, including enhanced hole‐transport resistance and extraction‐barrier effects, cannot be completely excluded for the observed EQE gradient.

The current spectral density (CSD) is measured in Figure 3f for devices with/without laser illumination. A CSD below 1 pA/ Hz^1/2^ CSD is obtained at 10 Hz for a large‐area device (0.49 cm^2^), due to the carrier‐blocking effect of NPB, which confirms the formed carrier‐injection barrier from NPB to n‐Si.

### 2.3 Si Narrowband Photodetector

**FIGURE 4:**
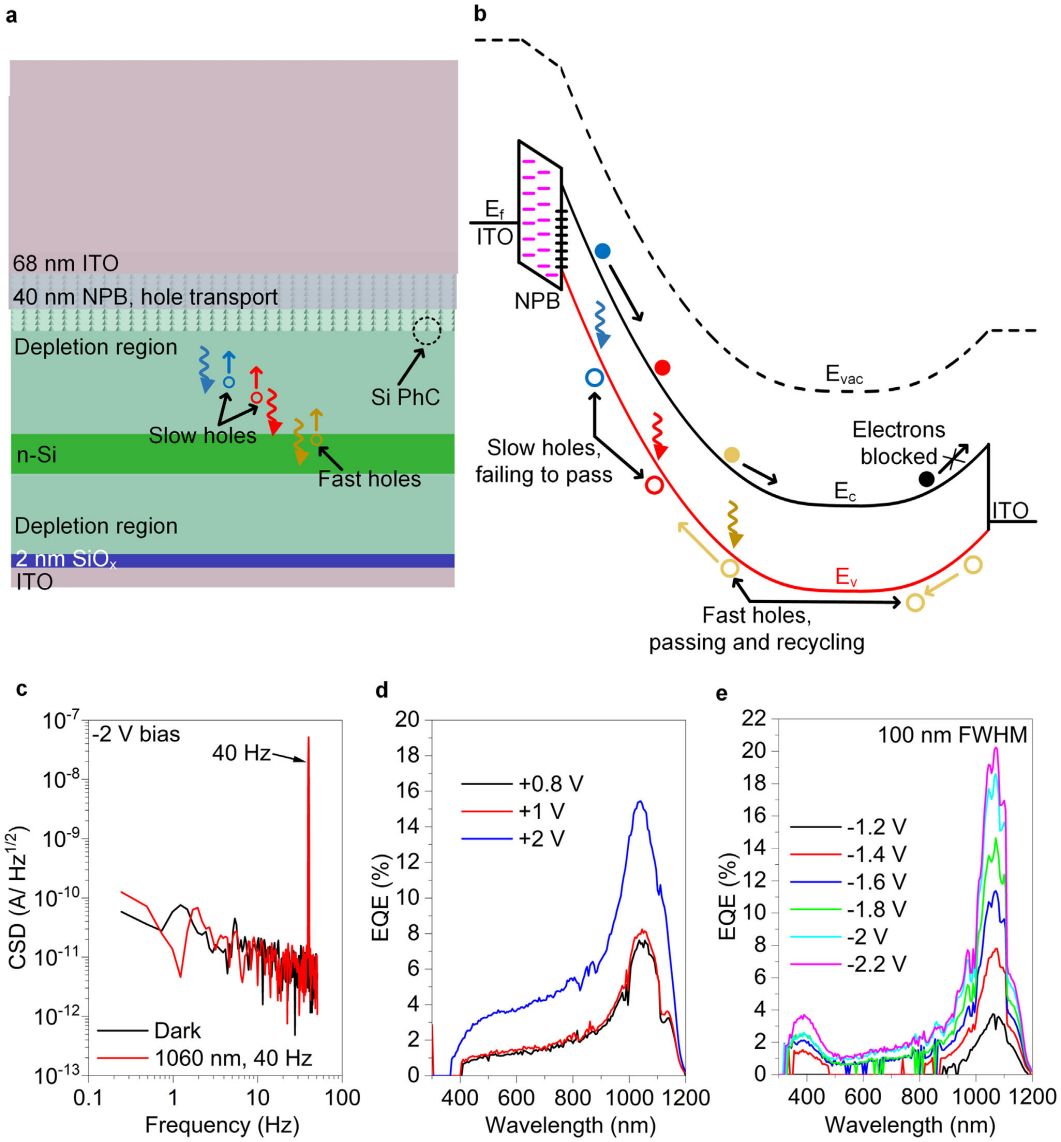
(a) Device structure, where a ∼40‐nm‐thick NPB layer is used to block slow carriers, and another ITO Schottky contact is located on the rear side to recycle fast carriers. (b) Energy band diagram, where the electron collection rate at the rear surface is markedly reduced due to the upward electron energy barrier, and those fast carriers, which succeed in passing through the thick NPB dielectric are recycled by the low hole injection barrier height from the rear ITO and n‐Si valence band. Note that the annealed ITO (rear contact) has a work function of 4.7–4.8 eV [9], and the unannealed ITO (top contact) has a work function of 4.3–4.6 eV [45]. (c) CSD with/without 1060 nm laser illumination. (d) EQE under positive biases (NPB–Si junction is forward‐biased), a narrowband collection is indicated. (e) EQE under negative biases, showing a narrowband collection.

A narrowband Si photodetector is demonstrated in Figure 4, which explores the slow‐carrier recombination effect and thereby illustrates both the potential application and universality of the observed phenomena. As discussed in Figure 3e, a ∼40 nm NPB layer impedes photocarrier transport, allowing only strong‐drift carriers to pass, thereby resulting in a photocarrier collection gradient. However, a broadband photoresponse is still observed due to electron flow enabled by the rear Al Ohmic contact, which contributes to photocurrent. In Figure 4a, the Al contact is replaced with another ITO Schottky contact, introducing an additional surface depletion region at the rear side [9]. The energy band diagram under reverse bias (top contact negatively biased) is shown in Figure 4b. The device exhibits two depletion regions at both the top and rear sides, which share the applied voltage drop. Namely, the voltage bias is dropped across the NPB layer, the NPB‐induced Si depletion region, and the rear‐side ITO‐induced Si depletion region. Thus, this energy band diagram can be referred to similarly to that of classic metal–semiconductor–metal photodetectors [51]. As illustrated in Figure 4b, there exists an electron barrier at the rear interface, further suppressing photoelectron collection from n‐Si to the rear‐side contact. Simultaneously, the work‐function alignment (also influenced by interface states) between the rear‐side ITO and n‐Si establishes a low‐energy hole barrier, permitting photohole injection from the rear‐side ITO Fermi level into the n‐Si valence band. Consequently, the EQE associated with slow photoholes that fail to traverse the 40 nm NPB layer is further suppressed due to reduced electron injection (also because the dual depletion regions share the voltage drop). In contrast, fast photoholes that successfully pass through the NPB layer are injected into the n‐Si valence band and recycled, thereby enabling a gain effect. The optical absorption for this device is shown in Figure S15.

A CSD of ∼10^−11^ A/Hz^1/2^ is identified in Figure 4c, at 10 Hz and −2 V bias. This noise current may arise from current fluctuations caused by asymmetric dual barriers (the top NPB–Si and bottom ITO–Si). When the NPB–Si junction is forward‐biased, the device exhibits a narrowband photoresponse (Figure 4d), explained by the fact that the rear‐side ITO Schottky contact (reverse‐biased) collects photocarriers generated near the rear surface (corresponding to long‐wavelength generation); similar results are reported elsewhere [52]. An FWHM greater than 200 nm is observed, with limited spectral selectivity. For example, the peak EQE at 1060 nm reaches ∼15% at +2 V, while the EQE at 700 nm is still ∼4.3%.

When the NPB–Si junction is reverse‐biased in Figure 4e, photocarriers generated in the 300–800 nm range are effectively filtered, with general EQE <2%. Meanwhile, fast photocarriers that pass through the NPB dielectric are amplified, yielding a peak EQE of ∼18.6% at 1070 nm (−2 V) and a narrowband spectral response with ∼100 nm FWHM, as suggested by carrier dynamics in Figure 4b. Note that the 1070 nm peak wavelength corresponds to the Si band edge. With the wide depletion region and increased surface recombination rate, these photocarriers generated at 1070 nm (distributed across the entire wafer thickness due to high optical absorption depth, Figure S2), achieve a high surface collection rate. It is potentially possible to realize a beyond‐band‐edge (e.g., 400–900 nm peak wavelength) Si narrowband response by reducing the Si wafer thickness (to condense the generation volume), integrating advanced light‐trapping designs (to spatially distinguish the generation from 400 to 900 nm), adopting appropriate device designs, and carefully controlling surface recombination, as a following‐up work.

The spectral responsivity and specific detectivity are plotted as a function of wavelength in Figure S16. Peak responsivity of ∼0.16 A/W and peak specific detectivity of ∼1.1 × 10^10^ Jones are obtained at 1070 nm (−2 V). Some key device performance metrics, including FWHM, EQE, peak responsivity and specific detectivity, are compared between our devices and the state‐of‐the‐art narrowband photodetectors (based on Si, perovskites, and organics) in Table S1 [14, 15, 52, 53, 54, 55, 56, 57, 58, 59, 60, 61, 62, 63]. Some perovskite‐ and organic‐based photodetectors demonstrate ultra‐narrow‐band colour‐selective responses with FWHM <80 nm, by strategies such as tuning the energy bandgap [14, 15]. In general, the ∼100 nm FWHM of our devices is competitively low among the Si‐based narrowband photodetectors [52, 60, 61, 62]. The specific detectivity of our devices at ∼10^10^ Jones is satisfactorily high among these narrowband photodetectors. This detectivity is mainly limited by the relatively low EQE due to carrier recombination and by the asymmetric barrier structure, which enhances noise current. Nevertheless, these performance metrics indicate the potential of this newly proposed slow‐carrier recombination effect in realizing high‐performance, multifunctional optoelectronic devices.

## 3 Conclusion

In this work, we demonstrate that semiconductor surfaces and certain dielectrics inherently tend to capture and fail weak‐drift charge carriers. This process is further engineered for tailoring spectral response in optoelectronic devices. These effects are validated by generating photocarriers within a wide depletion region and observing their surface collection efficiencies. We first verify the mechanism using a natural n‐Si surface with direct ITO contact, which reveals a wavelength‐dependent EQE gradient. This behavior is then extended to defective surfaces. Organic contacts of varying thickness are further employed to introduce significant barriers that distinguish photocarrier collection probabilities. Leveraging this understanding, we demonstrate a narrowband Si photodetector with an FWHM of ∼100 nm, enabled by recycling fast photocarriers and rejecting slow photocarriers. The proposed approach of converting conventional interfacial recombination, typically regarded as a loss mechanism, into a functional tool for spectral tuning holds significant promise for the design of next‐generation optoelectronic sensors.

## 4 Experimental Section

The experiment began with 4‐inch float‐zone wafers (n‐type, phosphorus‐doped, 3000 Ω·cm resistivity, ∼1.5 × 10^12^ cm^−3^ doping level, and double‐side polished). We first fabricated wave‐interference photonic crystals on Si; details of this process can be found elsewhere [64]. After fabricating these PhCs, the samples were exposed to the air to regrow native SiO_x_. A shadow mask was used to define the contact regions. ITO was sputtered using a Kurt. J. Lesker system with 100 W RF power, 20 sccm Ar flow, and a pressure of 2.5 mTorr. The sputtering rate for ITO was ∼1.5 nm/min. A 200 nm Al layer was sputtered as the rear‐side Ohmic contact (400 W RF power, 20 sccm Ar flow, and 5 mTorr chamber pressure, resulting in a rate of ∼7.2 nm/min). SiO_2_ was sputtered from a pure SiO_2_ target using 300 W power, 20 sccm Ar flow, and 5 mTorr pressure (∼1 nm/min). For the MoO_3_ sputtering process, a pure MoO_3_ target was used. The MoO_3_ films were deposited at room temperature with 240 W RF power, 20 sccm Ar/O_2_ gas flow with 5% O_2_ and 5 mTorr chamber pressure. The deposition rate was approximately 0.7 nm/min. NPB was thermally evaporated from the powder sources at a rate of ∼3 nm/min. For the devices in Figure 2, Figure 3, and Figure S12, NPB (or MoO_3_), ITO, SiO_2_, and Al were deposited in sequence, and no post‐deposition treatment was performed. For devices in Figure 4, 100 nm ITO was first deposited on the rear side of n‐Si, followed by annealing at 400°C in air for 30 min. NPB and ITO were then deposited on the front side without further treatment. A JEOL F200S/TEM system was used for HRTEM and EDS results. The current–voltage results were measured by a Keithley 4200 SCS semiconductor analyzer at room temperature. Optical reflection and transmission were measured using a UV–Vis spectrometer with an integrating sphere (Lambda PerkinElmer 1050). EQE was measured by a QE‐R system (Enlitech). Note that calibration errors from the reference cells, and the monochromator shift sometimes led to noisy EQE data points occurring at 800–1000 nm (e.g., 830 and 890 nm in Figure 2c,e; 830, 855, 890, 935, 965 nm in Figure 3e), which do not have physical significance. CSD measurements were performed using a current pre‐amplifier (Stanford Research SR570) and a fast Fourier transform (FFT) spectrum analyzer (Stanford Research Systems, SR770), where a 1060 nm fibre‐coupled laser (Qphotonics, QFBGLD‐1060‐10BTF) was used for illumination. The laser was mounted in a Thorlabs LM14S2 laser mount, which was connected to an LDC 210C diode laser driver (Thorlabs) and TED 200C temperature controller (Thorlabs). A function generator (Hewlett Packard 33120A) was used to modulate the laser frequency.

### 4.1 Statistical Analysis

We conducted all experiments more than three times to confirm reproducibility. The electric field is normalized in Figure 3d, while all other results are shown as directly measured from the instruments.

## Author Contributions

Y. Zhang observed the phenomena, conceived and conducted experiments, characterized the devices, collected and analyzed data, and prepared and revised the draft of the manuscript. H. Wang deposited the NPB layers, assisted with the characterizations, conducted the FDTD simulation, and edited the draft. Z. Liu, Y. Zhong, and Z. H. Lu assisted with the contact deposition. N. P. Kherani discussed the results, reviewed and edited the final manuscript, and supervised the research. All authors reviewed the draft.

## Conflicts of Interest

The authors declare no conflicts of interest.

## Supporting information




**Supporting File**: advs76887‐sup‐0001‐SuppMat.pdf.

## Data Availability

The data that support the findings of this study are available from the corresponding author upon reasonable request.
