# Supplementary material for: Engineering Slow‐Carrier Interfacial Recombination Enables Tailored Spectral Response
Source: Adv Sci (Weinh). 2026 Jul 31:e76887. Online ahead of print. doi: 10.1002/advs.76887 (PMC13426101; doi:10.1002/advs.76887)
Supplement: Supplementary file 1 — Supporting File: advs76887‐sup‐0001‐SuppMat.pdf. [file ADVS-9999-e76887-s001.pdf]

# Supporting Information

**Engineering Slow-Carrier Interfacial Recombination Enables Tailored Spectral Response**

Yibo Zhang,*[,1] Haozhe Wang,[1] Zeke Liu,[2] Yun Zhong,[2] Zheng-Hong Lu,[2] and Nazir P. Kherani*[,1,2]

[1]The Edward S. Rogers Sr. Department of Electrical and Computer Engineering, University of Toronto, 10 King's College Road, Toronto, Ontario M5S 3G4, Canada
[2]Department of Materials Science and Engineering, University of Toronto, 184 College Street, Toronto, Ontario M5S 3E4, Canada

*Emails: Y. Z. (yibojhhk.zhang@mail.utoronto.ca); N. P. K. (nazir.kherani@utoronto.ca)

## 1. Depletion region width as a function of voltage.

The capacitance–voltage (C–V) results of the unannealed ITO/n-Si (~$1.5\times10^{12}$ cm$^{-3}$ doping level)/Al device are shown in Figure S3a, and were previously reported in Ref.[1]. Here, the C–V data are reanalyzed to experimentally determine the depletion region width using the following equation:

$$W = \frac{\varepsilon_{si} A}{C} \quad (1)$$

Where $W$ is the depletion region width, $C$ is the capacitance, $A$ is the effective device area, $\varepsilon_{Si}$ is the Si permittivity. This equation assumes a parallel-plate capacitor model for the wide depletion region. The extracted depletion width is plotted as a function of voltage on both logarithmic (Figure S3b) and linear (Figure S3c) scales. The presence of interface states, series resistance, and the non-ideal parallel-plate-capacitor assumption mean that the TCAD model and C–V experimental results cannot match exactly in determining the depletion width. Nevertheless, a depletion region width of approximately 29–40 μm is experimentally obtained for reverse bias magnitude greater than 1.5 V (Figure S3c), in good agreement with the TCAD simulations.[1]

## 2. Quantity of the interface-state change by effective Schottky barrier height measurement.

The current density–voltage (J–V) curves are measured in Figure S4, for ITO/n-Si/Al devices with/without a $SiO_2$ layer atop ITO. ~3 Ω·cm n-Si (~$1.5\times10^{15}$ cm$^{-3}$ doping level) integrated with photonic crystals was used for the devices. Note that the ITO–Si samples were annealed in air before depositing the $SiO_2$ layer to passivate the interface and enlarge the effective Schottky barrier height $\Phi_B$,[2] allowing better visualization of the defects introduced by the $SiO_2$ layer. By extracting the $\Phi_B$ using Cheung's method (Figure S5),[3] the $\Phi_B$ decreases from ~0.73 eV to ~0.6 eV after depositing the $SiO_2$ layer atop ITO. The reduced effective $\Phi_B$, resulting from the Fermi-level pinning effect, indicates the introduced defects at the ITO/n-Si interface during $SiO_2$ deposition.

## 3. Spectral responsivity and specific detectivity

The spectral responsivity and specific detectivity are shown in Figure S16. The spectral responsivity $R_\lambda$ (Figure S16a) is calculated by the following equation:

$$R_\lambda = EQE \times \lambda \times \frac{e}{hc} \tag{2}$$

where $\lambda$ is the wavelength, $e$ is the elementary charge, $h$ is Plank's constant, and $c$ is the vacuum light speed. The specific detectivity D* (Figure S16b) is calculated by the following equation:

$$D^* = \frac{R_\lambda \sqrt{A}}{S_n} \tag{3}$$

where $S_n$ is the noise spectral density.

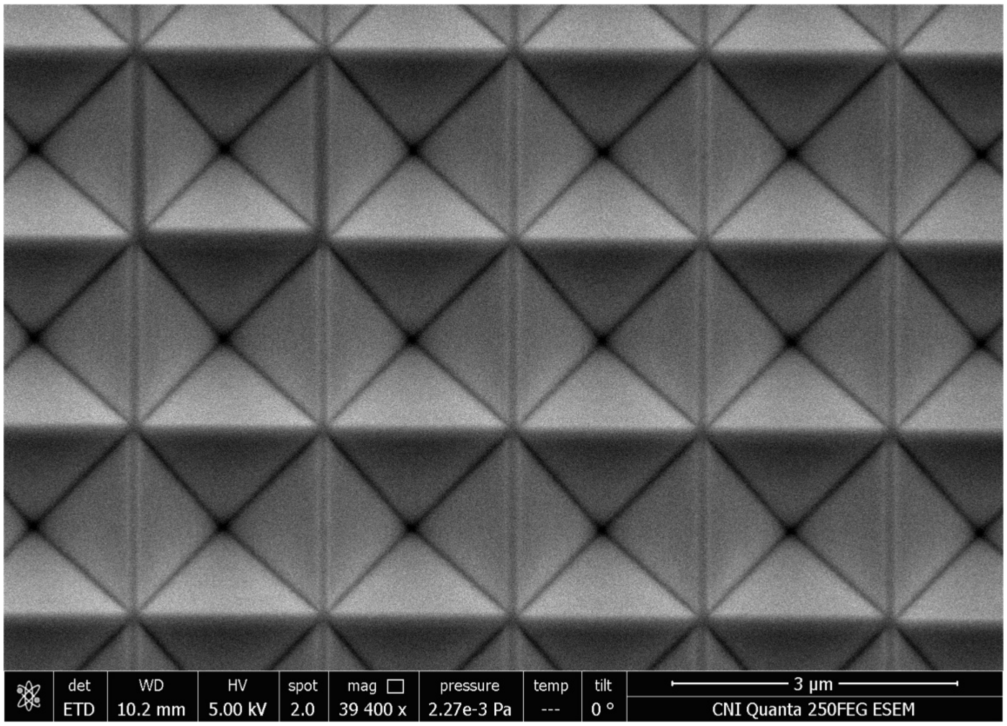


**Figure S1**. Scanning electron microscopy image for ITO/NPB/Si PhC devices.

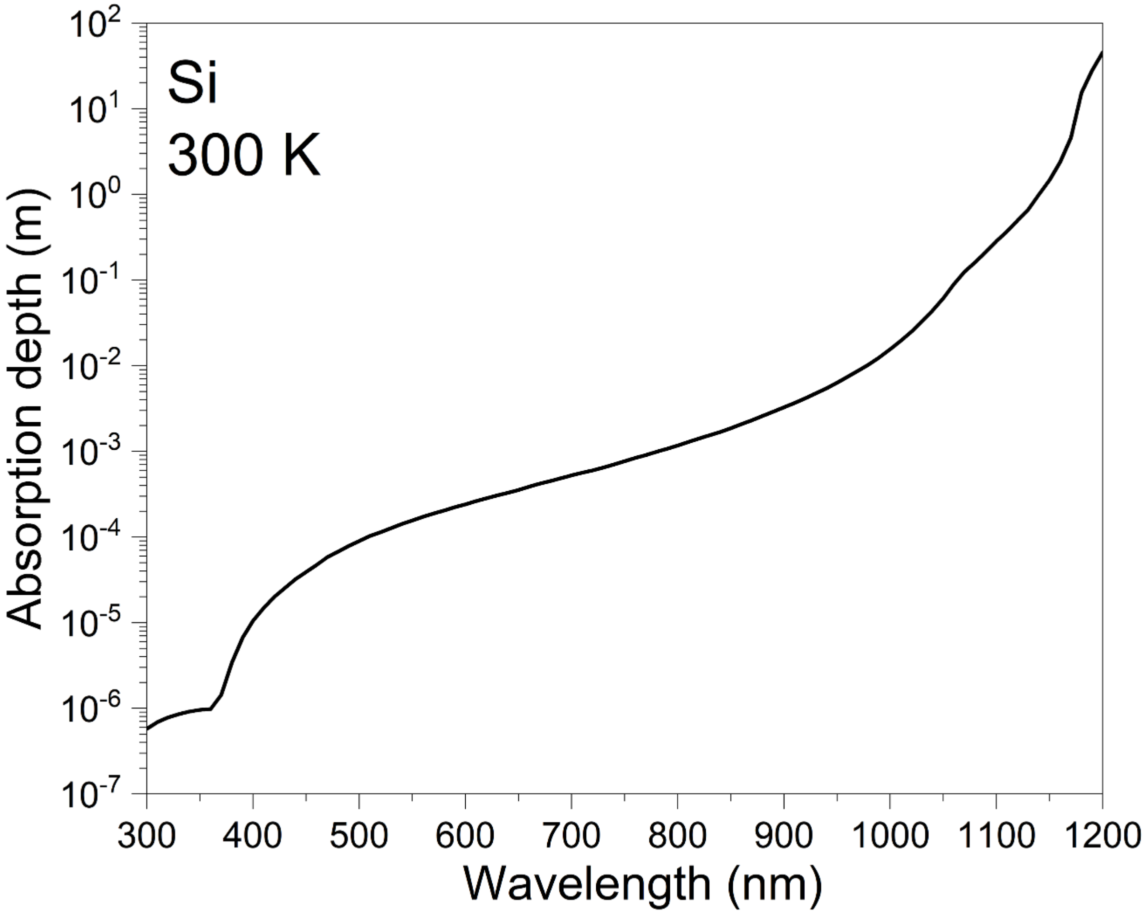


**Figure S2**. Optical absorption depth of single-crystalline Si at 300 K.

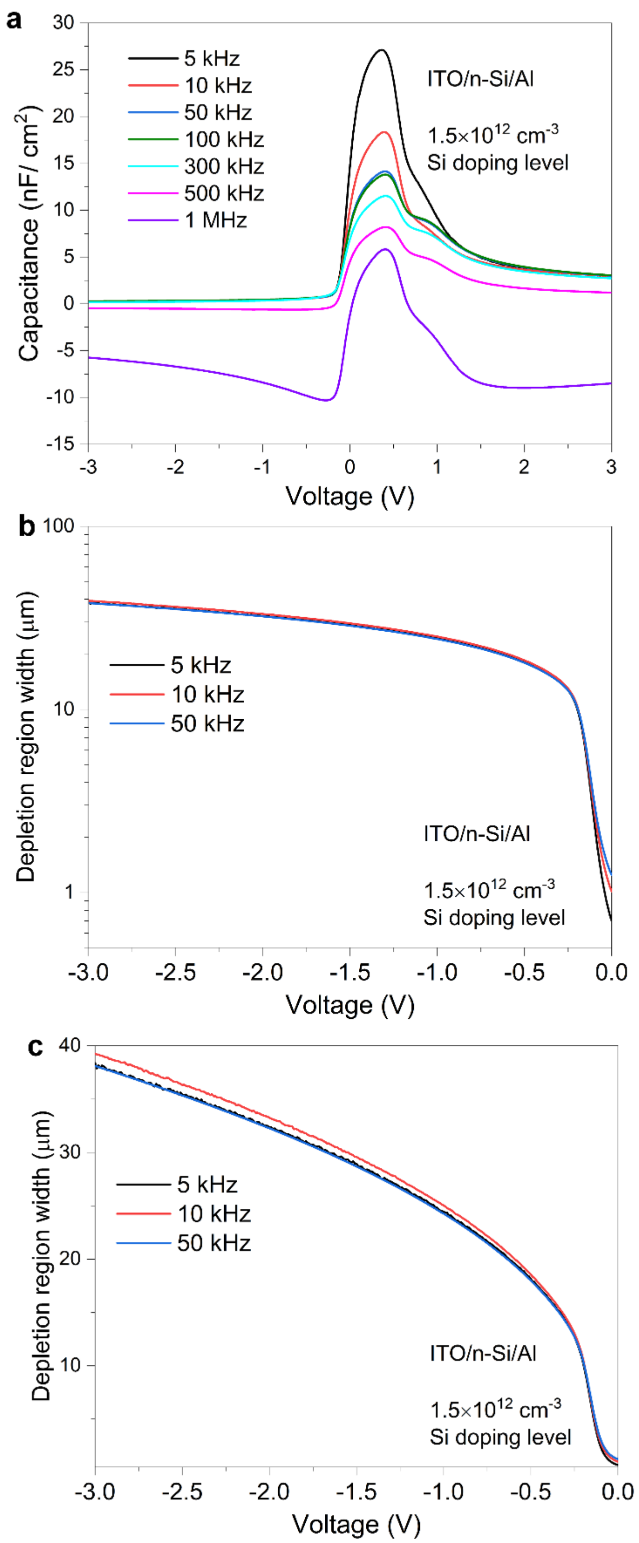


**Figure S3**. (**a**) C–V data for ITO/n-Si (~$1.5\times10^{12}$ $cm^{-3}$ doping level)/Al structure, at varying frequencies. The C–V data were previously reported in Ref.[1] and are reanalyzed here to extract the depletion region width as a function of reverse bias. (**b**) Depletion region width as a function of applied voltage, on a logarithmic scale. (**c**) Depletion region width as a function of applied voltage, on a linear scale.

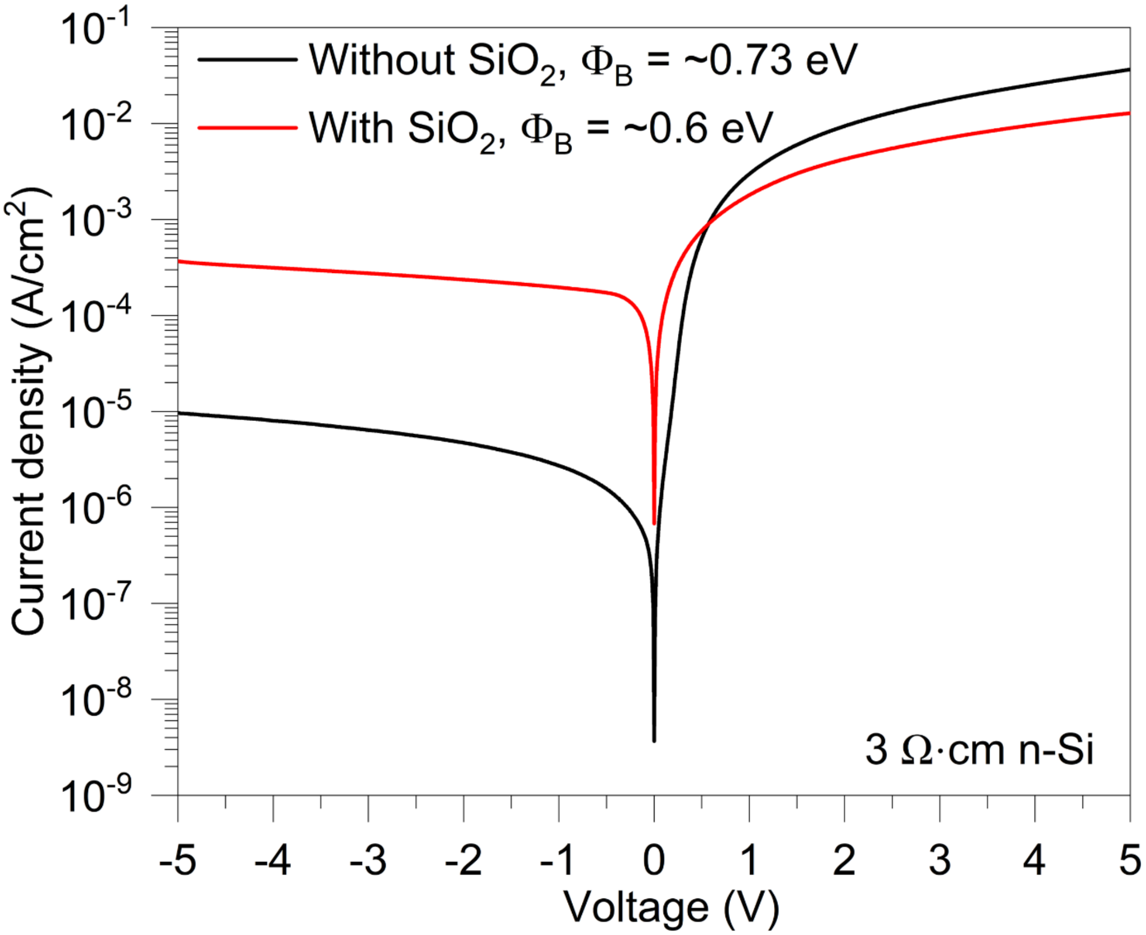


**Figure S4**. Current densty–voltage (J–V) curves for ITO/n-Si/Al devices with/without $SiO_2$.

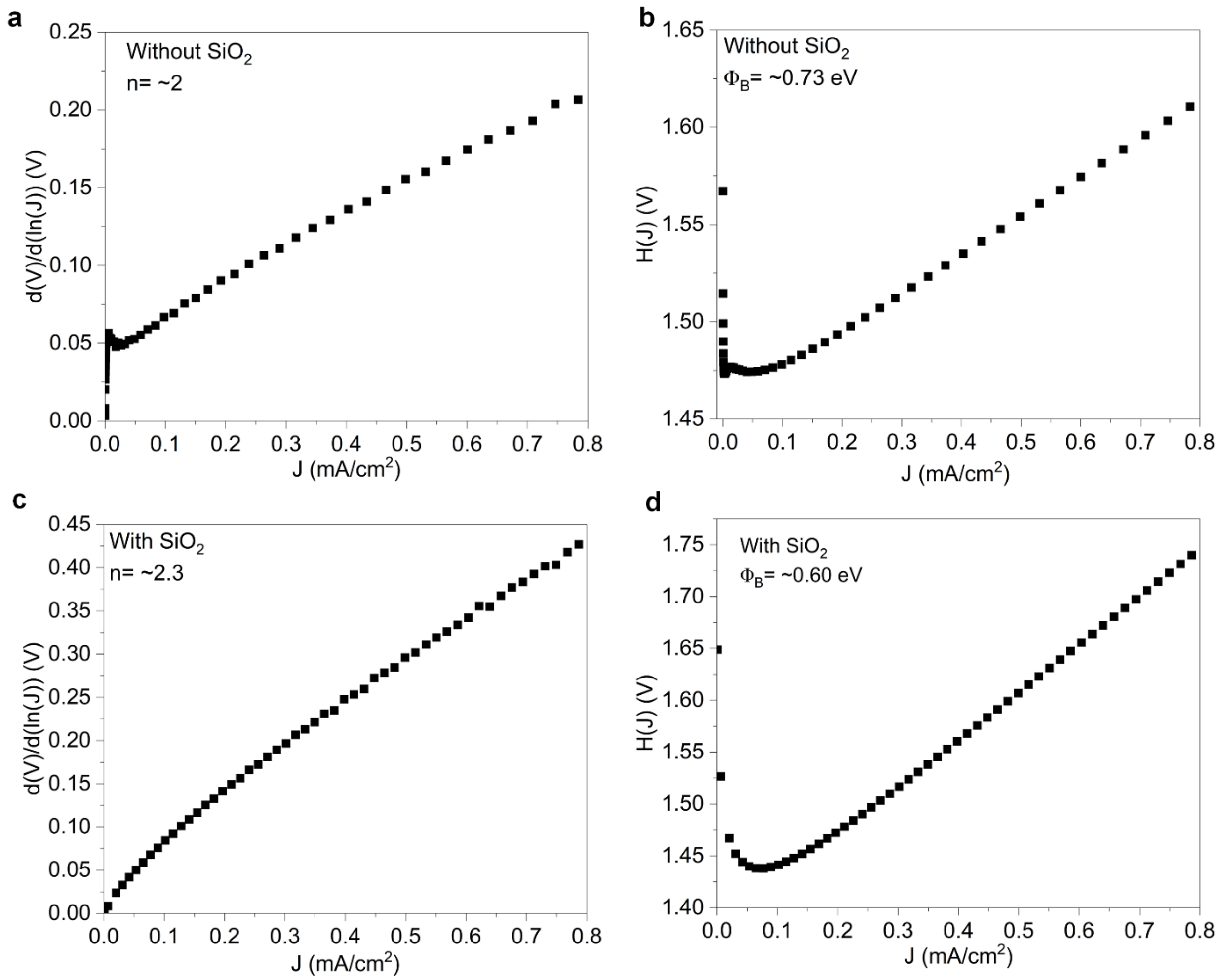


**Figure S5**. Schottky barrier height fitting, for ITO/n-Si/Al devices with/without $SiO_2$. (**a**) d(V)/d(In(J)) as a function of J, for devices without $SiO_2$. (**b**) H(J) as a function of J, for devices without $SiO_2$. (**c**) d(V)/d(In(J)) as a function of J, for devices with $SiO_2$. (**d**) H(J) as a function of J, for devices with $SiO_2$.

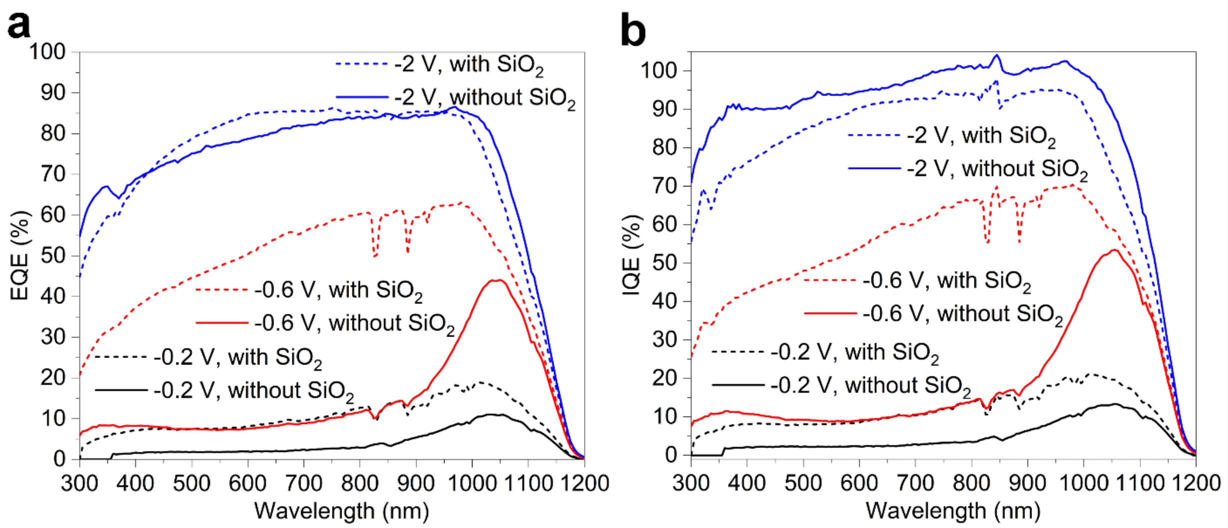


**Figure S6**. EQE and IQE comparison for devices with/without $SiO_2$, at different biases. (**a**) EQE. (**b**) IQE.

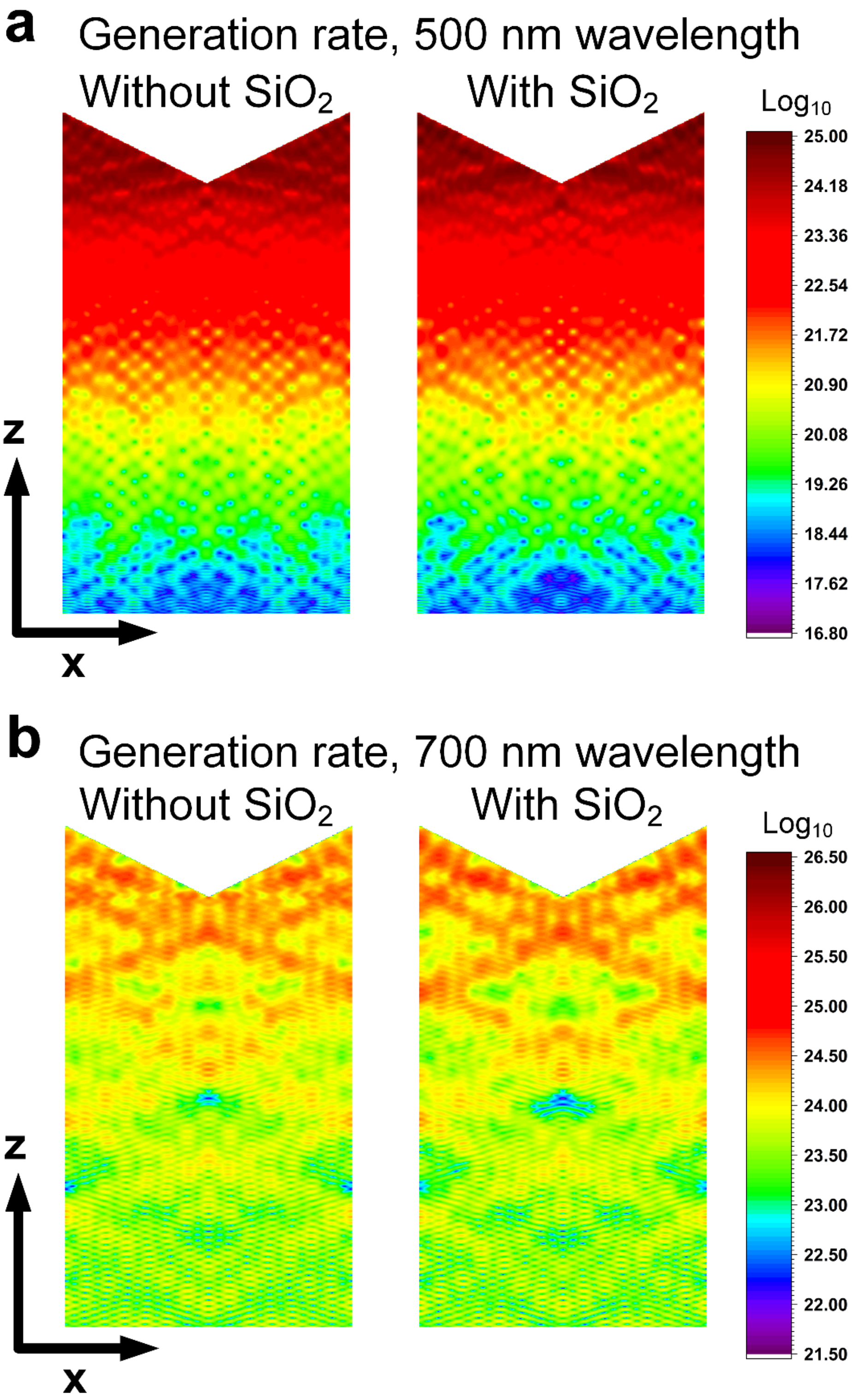


**Figure S7**. FDTD simulations showing the photogeneration rate for 10 nm ITO/Si photonic crystal structures, with/without $SiO_2$ at the top. (**a**) Illuminated by 500 nm wavelength light. (**b**) Illuminated by 700 nm wavelength light. The simulated area is 2 μm × 10 μm. Note: The x and z axes are not plotted to scale.

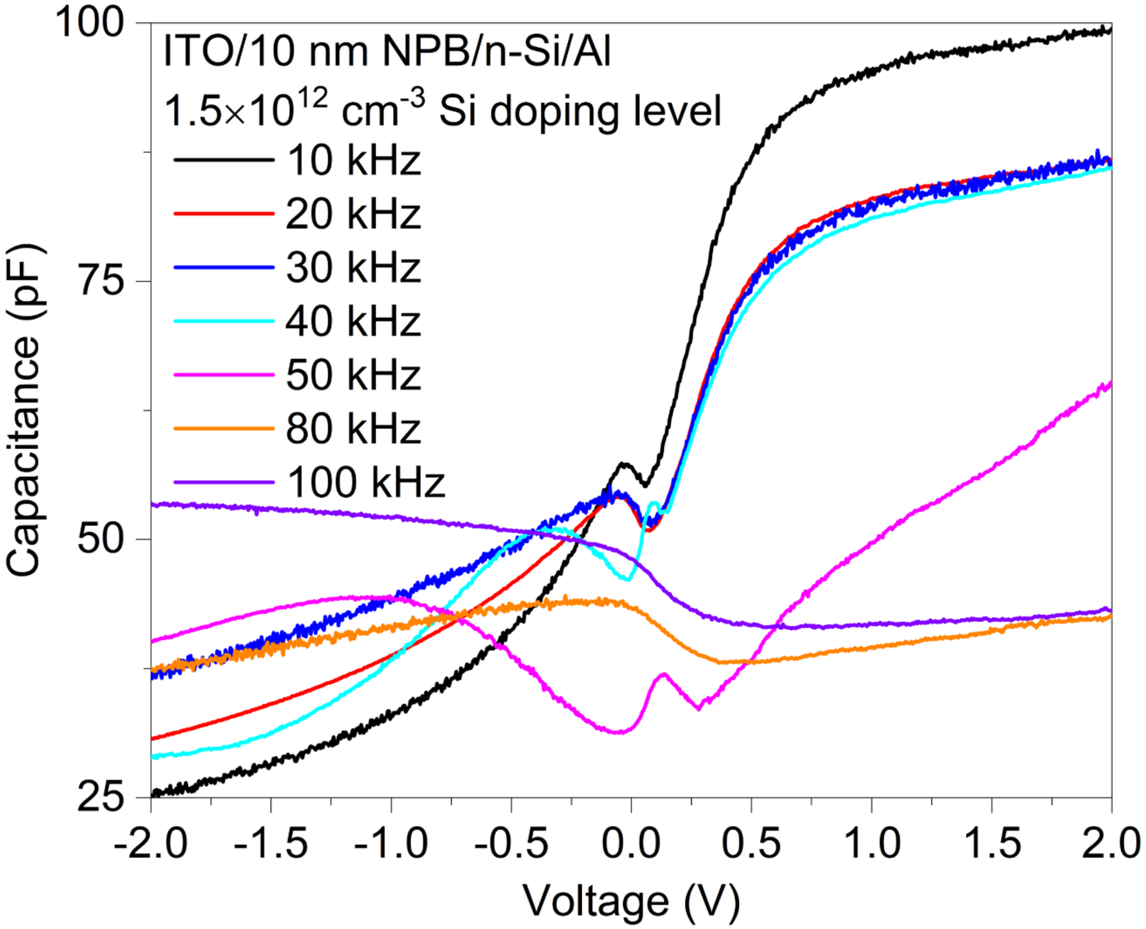


**Figure S8**. C–V curves for ITO/10 nm NPB/n-Si (~$1.5\times10^{12}$ $cm^{-3}$ doping level)/Al device, at varying frequencies.

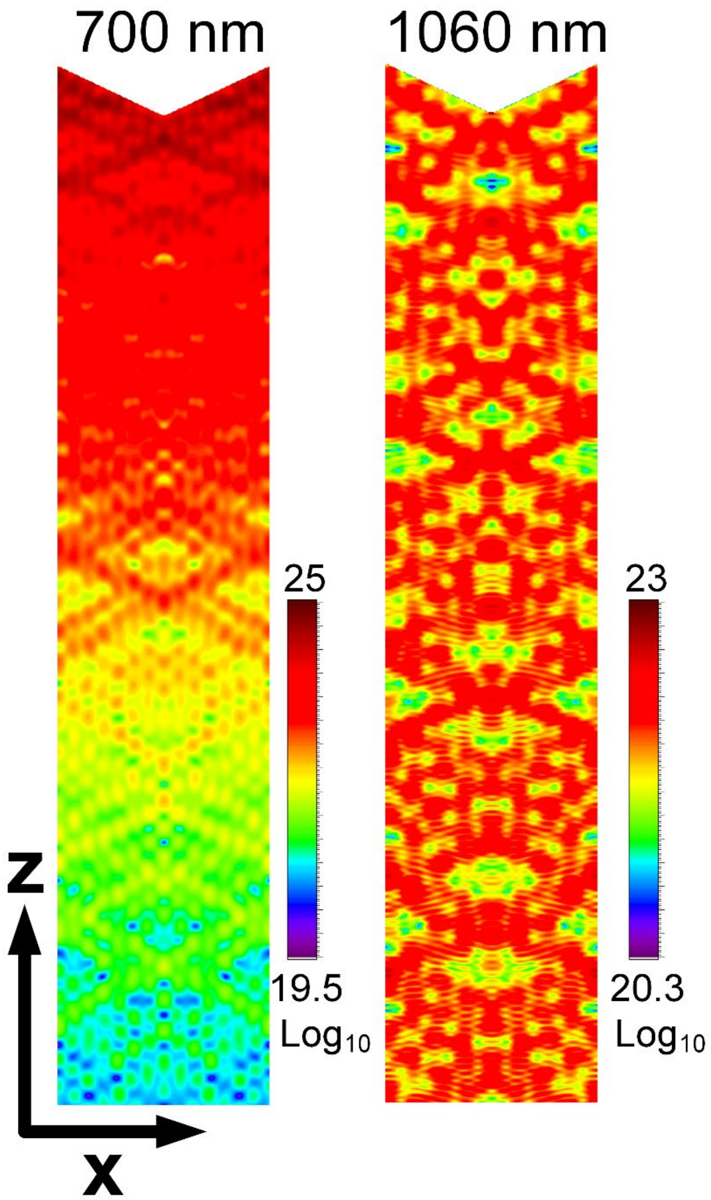


**Figure S9**. FDTD-simulated optical generation profiles for 700 nm and 1060 nm illumination.

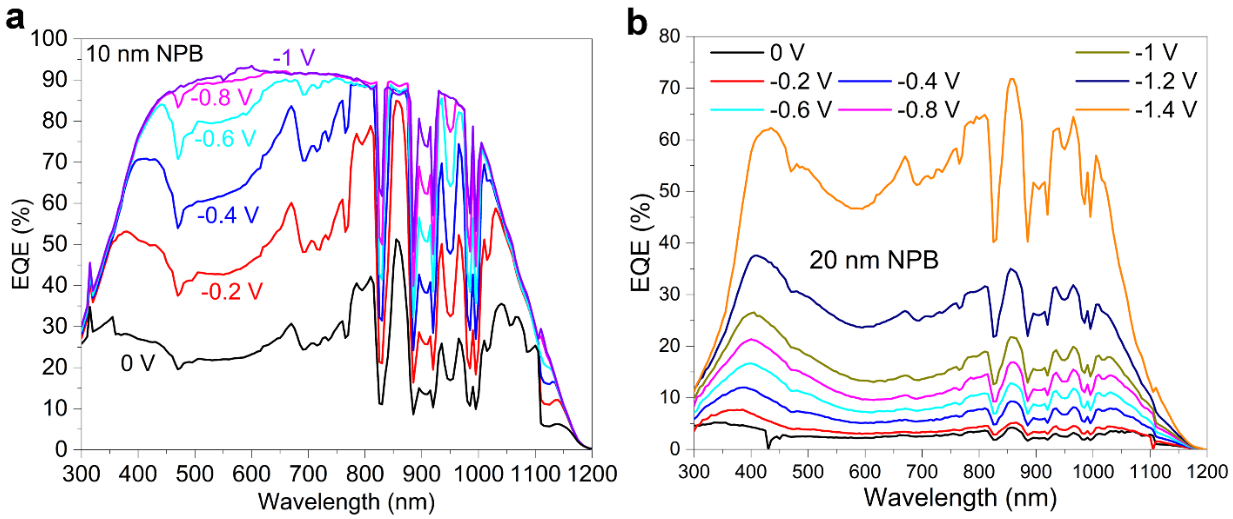


**Figure S10**. EQE results for devices with varying NPB thicknesses. (**a**) 10 nm NPB. (**b**) 20 nm NPB.

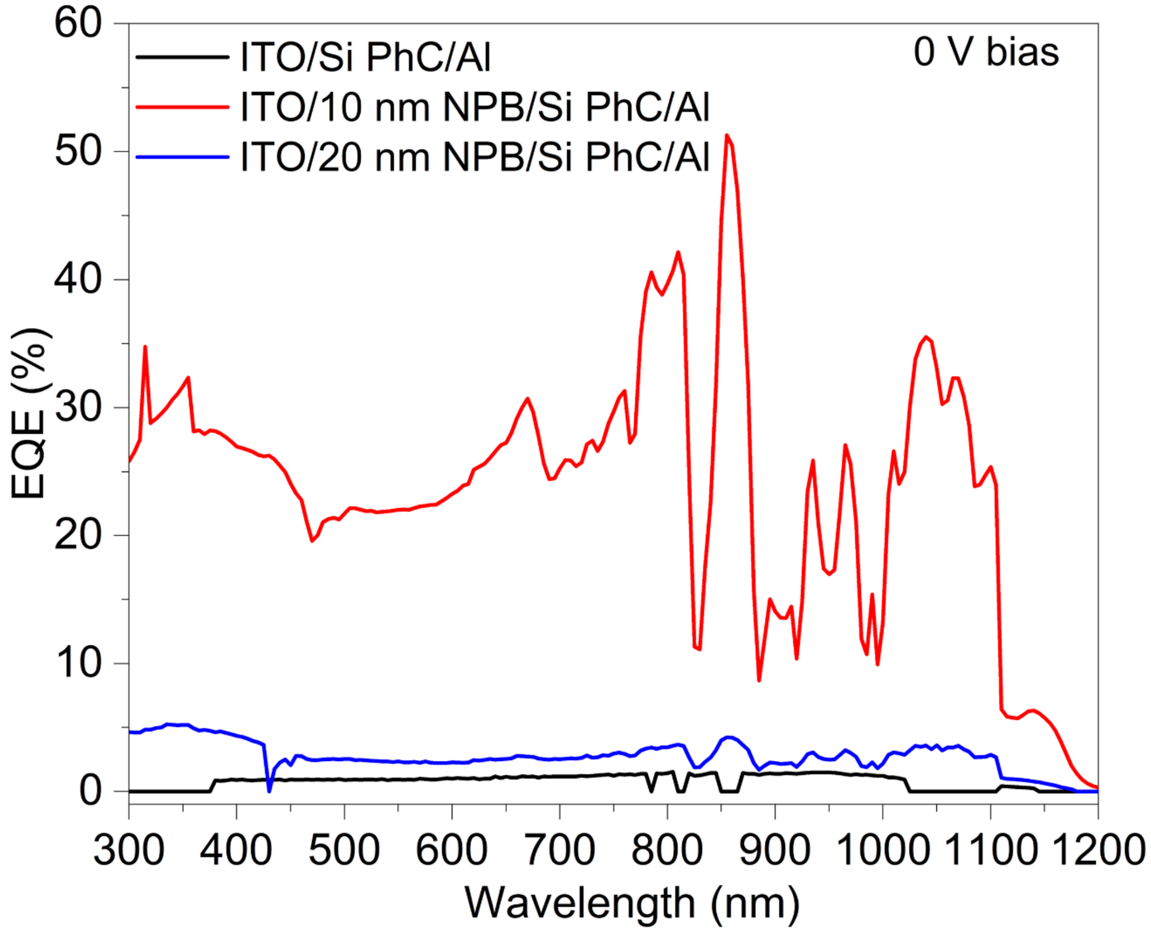


**Figure S11**. EQE comparison at 0 V bias, between ITO/Si PhC/Al, and ITO/NPB/Si PhC/Al devices.

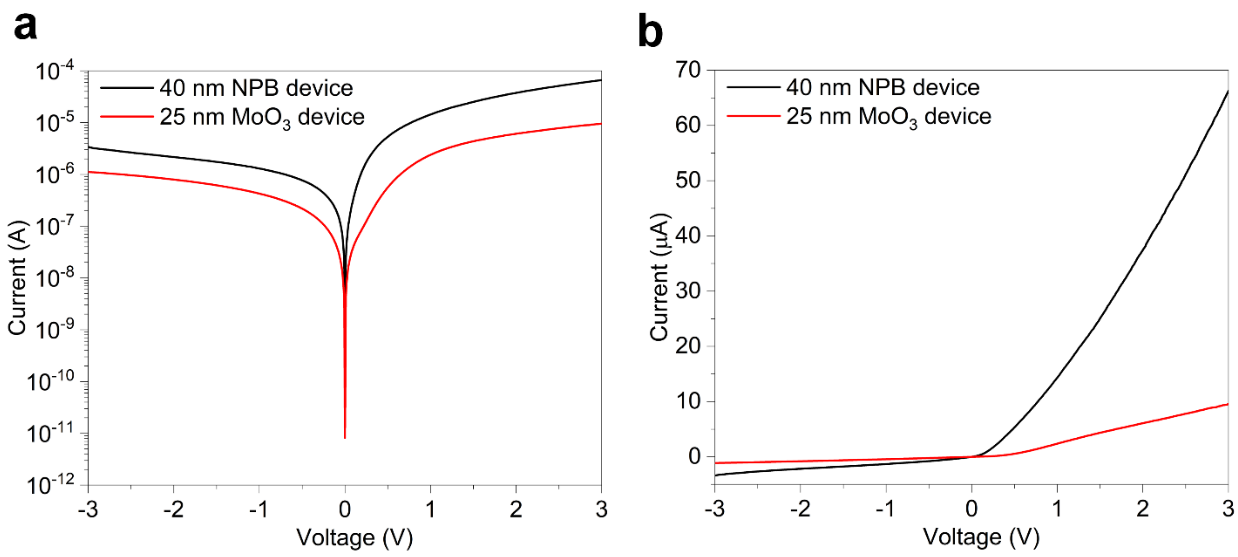


**Figure S12.** Current–voltage curves for NPB and $MoO_3$ devices. (**a**) Logarithmic scale. (**b**) Linear scale.

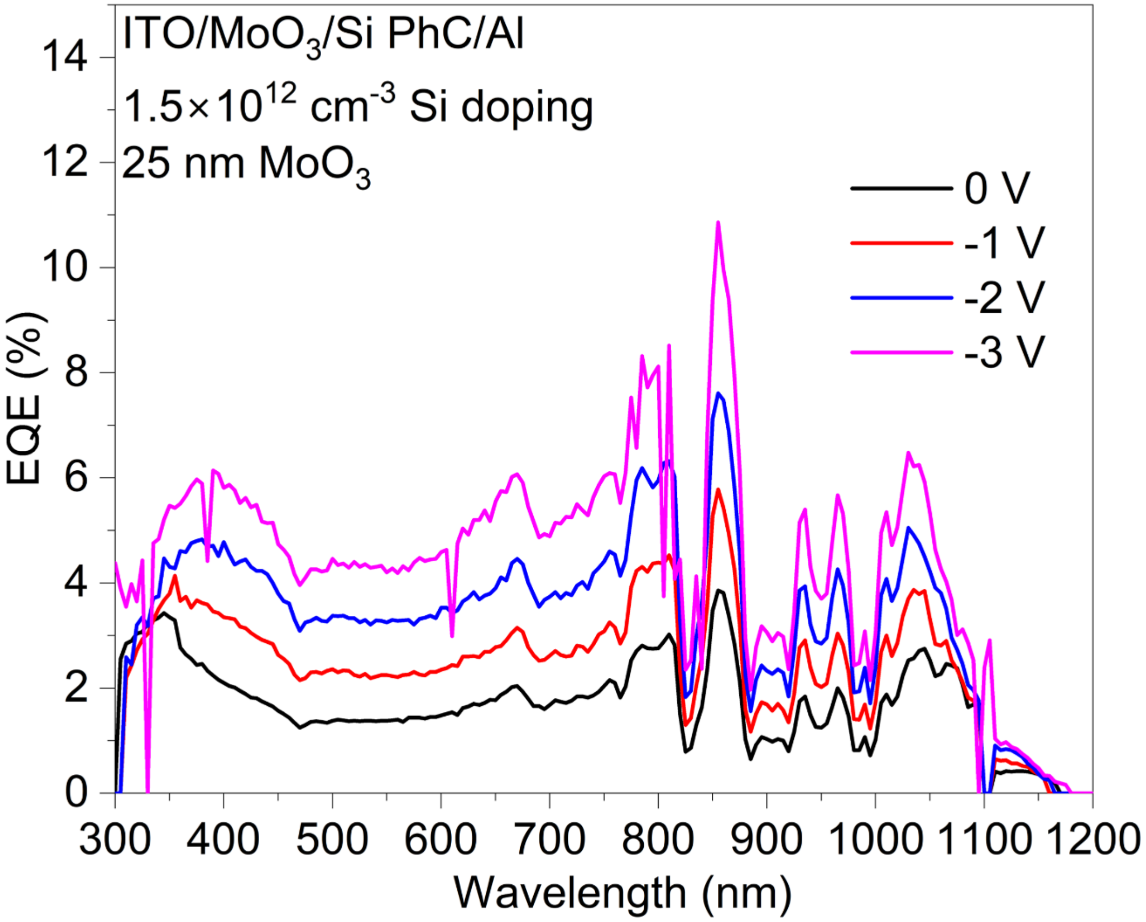


**Figure S13**. EQE results for a control device with 25 nm $MoO_3$ replacing NPB, for enhanced hole-transport resistance.

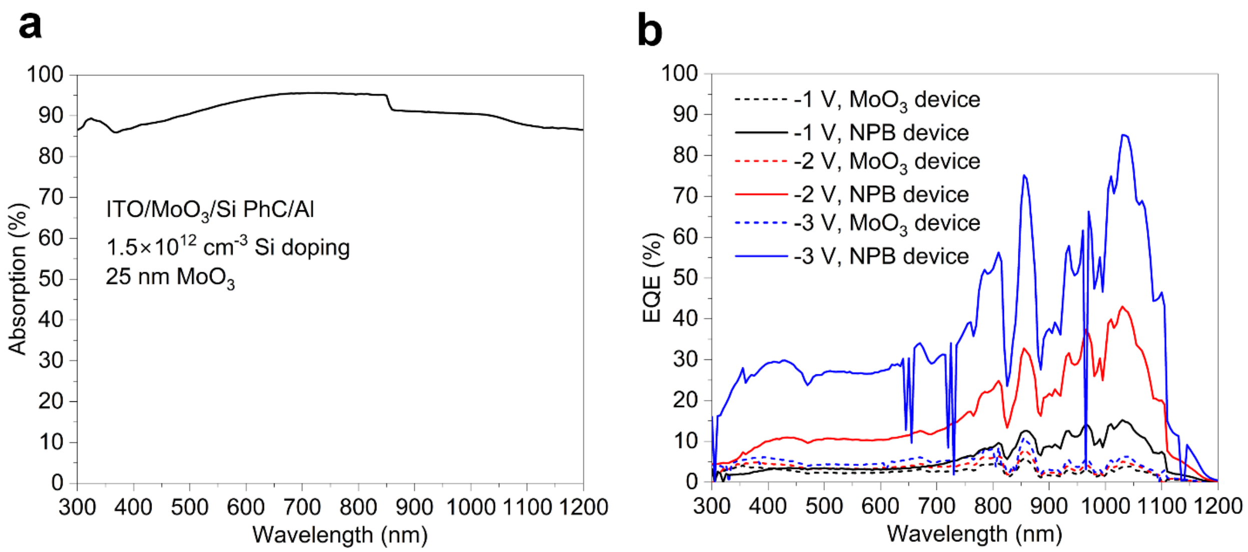


**Figure S14.** (**a**) Optical absorption of ITO/$MoO_3$/Si PhC/Al device. (**b**) EQE comparison between 40-nm NPB and 25-nm $MoO_3$ devices.

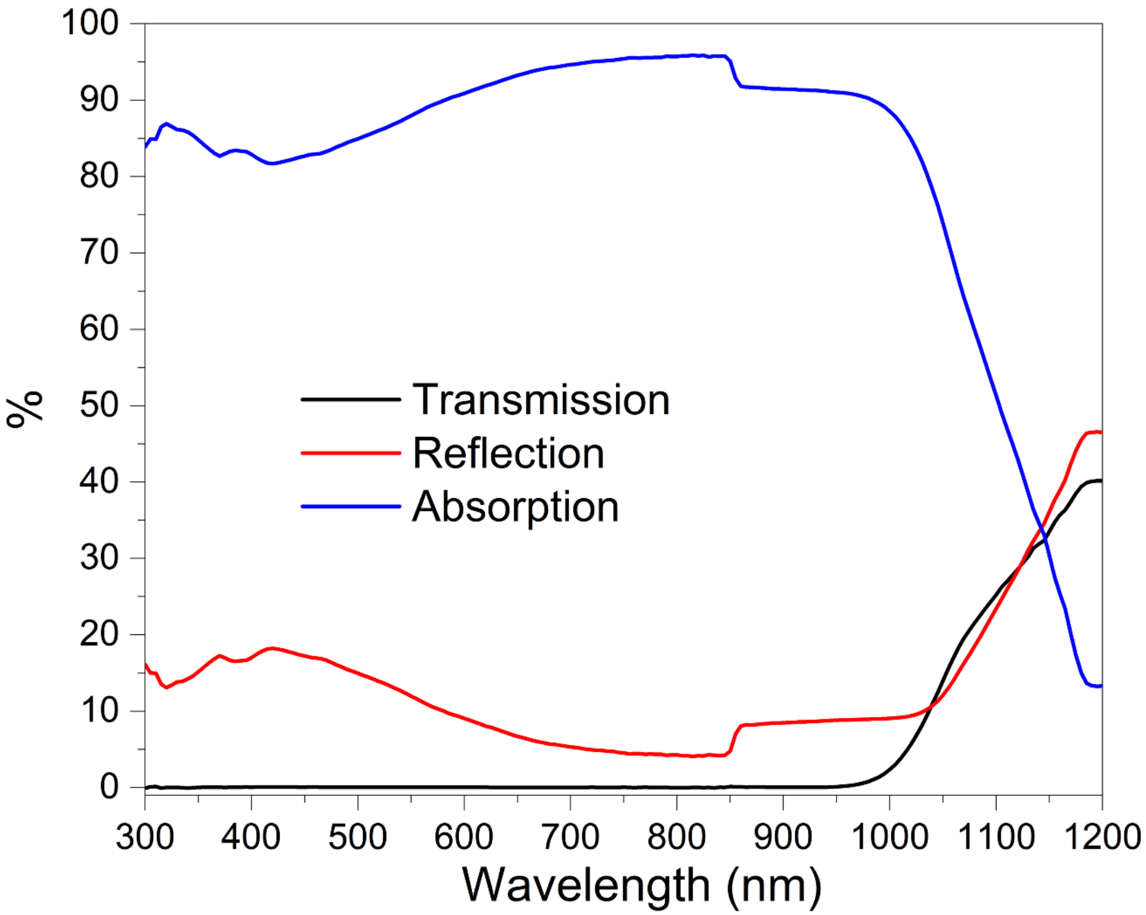


**Figure S15**. Reflection, transmission and absorption for ITO/NPB/Si PhC/ITO devices.

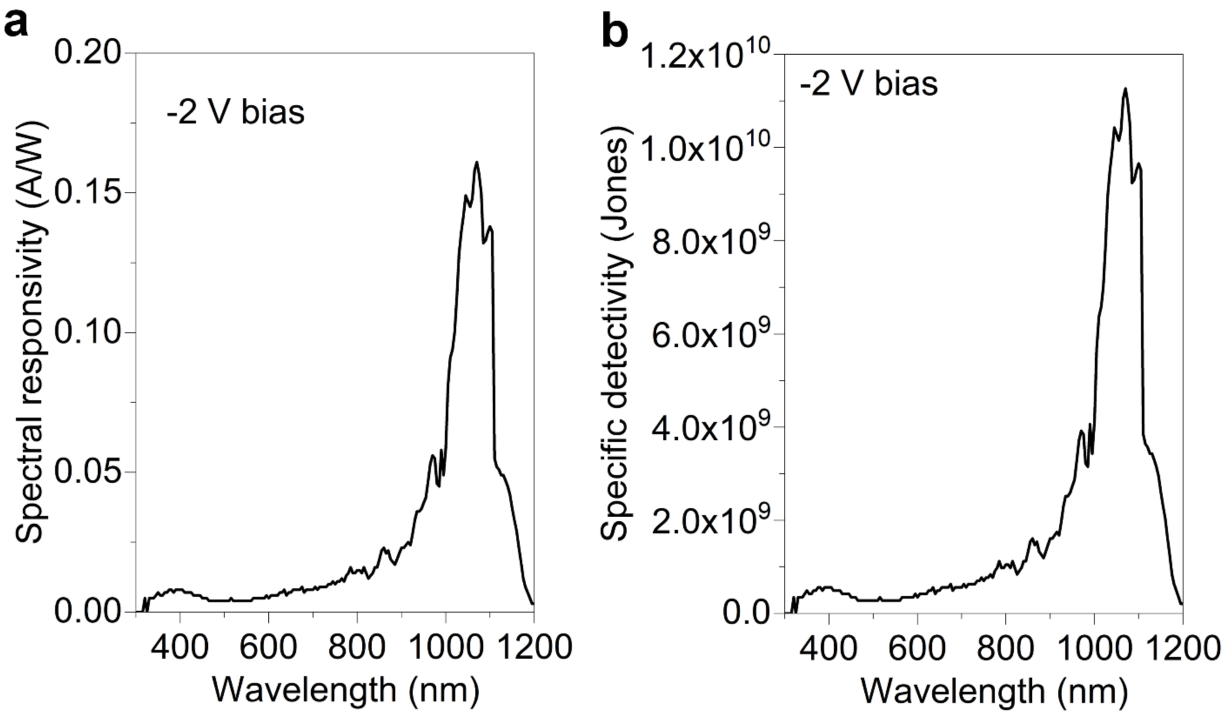


**Figure S16**. Spectral responsivity (**a**) and specific detectivity (**b**) as a function of wavelength.

**Table S1.** Performance comparison between our devices and the state-of-the-art organics-, perovskite-, and Si-based narrowband photodetectors, in terms of peak wavelength, FWHM, EQE, responsivity and specific detectivity.

| Materials | Wavelength (nm) | FWHM (nm) | EQE (%) | Responsivity (A/W) | Detectivity (Jones) | References |
|---|---|---|---|---|---|---|
| Organics | 860 | 50 | 65 | N/A | $10^{13}$ | Ref. 4 |
| Organics | 810–1550 | 36 | 20 | N/A | $10^{11}$ | Ref. 5 |
| Organics | 1080 | 56 | 53 | 0.46 | $2.34\times10^{12}$ | Ref. 6 |
| Organics | 785 | 63 | N/A | 0.024 | $10^{9}$ | Ref. 7 |
| Organics | 680 | 54 | 5840 | 32 | $2.6\times10^{13}$ | Ref. 8 |
| Perovskite | 450–650 | 80 | 12 | N/A | $2\times10^{11}$ | Ref. 9 |
| Perovskite | 400–700 | 20 | 1.6 | N/A | $2\times10^{10}$ | Ref. 10 |
| Perovskite | 810 | 33 | $10^{4}$ | 275 | $10^{13}$ | Ref. 11 |
| Perovskite | 800 | 30 | 17 | 0.0637 | $1.27\times10^{12}$ | Ref. 12 |
| Perovskite | 560–660 | 38 | 80 | 0.41 | $3.7\times10^{11}$ | Ref. 13 |
| Si | 1060 | 107 | 135 | N/A | $10^{11}$ | Ref. 14 |
| Si | 1080 | 101 | N/A | N/A | $1.84\times10^{12}$ | Ref. 15 |
| Si | 1120 | 83 | N/A | N/A | $8.7\times10^{11}$ | Ref. 16 |
| Si | 1060 | N/A | N/A | 0.62 | $1.04\times10^{11}$ | Ref. 17 |
| Si | 1070 | 100 | 18.6 | 0.16 | $1.1\times10^{10}$ | This work |

References


[1] Y. Zhang, S. Almenabawy, N. P. Kherani, Haynes-Shockley experiment analogs in surface and optoelectronics: Tunable surface electric field extracting nearly all photocarriers. *Sci. Adv.* **2023**, *9*, eadg2454.

[2] Y. Zhang, J. Y. Y. Loh, A. G. Flood, C. Mao, G. Sharma, N. P. Kherani, Ultra-Sensitive Cubic-ITO/Silicon Photodiode via Interface Engineering of Native SiOx and Lattice-Strain-Assisted Atomic Oxidation. *Adv. Funct. Mater.* **2022**, *32,* 2109794.

[3] S. K. Cheung, N. W. Cheung, *Appl. Phys. Lett.* **1986**, *49*, 85-87.

[4] B. Xie, R. Xie, K. Zhang, Q. Yin, Z. Hu, G. Yu, F. Huang, Y. Cao, Self-filtering narrowband high performance organic photodetectors enabled by manipulating localized Frenkel exciton dissociation. *Nat. Commun.* **2020**, *11*, 2871.

[5] S. Bernhard, A. Mischok, J. Benduhn, O. Zeika, S. Ullbrich, F. Nehm, M. Böhm, Organic narrowband near-infrared photodetectors based on intermolecular charge-transfer absorption. *Nat. Commun.* **2017**, *8,* 15421.

[6] T. Wei, G. Madhaiyan, L. Lai, Y. Hsiao, J. Wu, C. Liao, C. Hou, J. Shyue, Y. Chang, Bulk-heterojunction adjustment enables a self-filtering organic photodetector with a narrowband response. *ACS Appl. Mater. Interfaces* **2022**, *14,* 38004-38012.

[7] S. Siddhartha, A. Shukla, S. Lo, Ebinazar B. Namdas, Narrow band organic photodiode with photoresponse at 808 nm for photoplethysmography. *Adv. Photon. Res.* **2024**, *5,* 2400003.

[8] S. Gajendra, C. Chu, F. Chen, High-Performance Self-Filtering Organic Photodetectors with Photomultiplication Narrowing. *Adv. Opt. Mater.* **2024**, *12,* 2400662.

[9] Q. Lin, A. Armin, P. L. Burn, P. Meredith, Filterless narrowband visible photodetectors. *Nat. Photon.* **2015**, *9*, 687-694.

[10] Y. Fang, Q. Dong, Y. Shao, Y. Yuan, J. Huang, Highly narrowband perovskite single-crystal photodetectors enabled by surface-charge recombination. *Nat. Photon.* **2015**, *9*, 679-686.

[11] S. Makhsud I, M. A. Haque, M. Savoie, A. L. Abdelhady, N. Cho, I. Dursun, U. Buttner, E. Alarousu, T. Wu, O. M. Bakr, Perovskite photodetectors operating in both narrowband and broadband regimes. *Adv. Mater.* **2016**, *37,* 8144-8149.

[12] W. Jian, S. Xiao, W. Qian, K. Zhang, J. Yu, X. Xu, G. Wang, S. Zheng, S. Yang, Self-driven perovskite narrowband photodetectors with tunable spectral responses. *Adv. Mater.* **2021**, *33,* 2005557.

[13] O. Z. Ying, S. Nie, G. Vega, M. C. Lai, A. Jiménez-Solano, C. S. Huang, H. Wang, Resonant cavity effect for spectrally tunable and efficient narrowband perovskite photodetectors. *ACS Photonics* **2025**, *12,* 4119-4129.

[14] L. Wang, H. Luo, H. Zuo, J. Tao, Y. Yu, X. Yang, M. Wang, J. Hu, C. Xie, D. Wu, L. Luo, Highly Sensitive Narrowband Si Photodetector With Peak Response at Around 1060 nm. *IEEE Trans. on Electron Devices* **2020**, *67*, 3211-3214.

[15] J. Xing, Y. Liu, Y. Gao, M. Wang, G. Zhang, C. Wu, Z. Zhu, Deep-Level Impurity Enabled Planar Filterless Narrowband Near-Infrared Si Photodetector. *IEEE Electron Device Lett.* **2024**, *45,* 440-443.

[16] M. Liang, C. Fu, Y. Li, B. Yu, F. Shi, Y. Gao, S. Long, F. Liang, L. Luo, Study of Ultranarrowband Silicon Nanowire Array Photodetector with Peak Spectral Responsivity at 1120 nm. *ACS Nano.* **2025**, *19,* 32848-32857.

[17] B. Park, S. Beak, J. Yang, S. Hwang, J. Kwon, J. Yoon, S. Kwon, Y. Kim, Tellurium/Silicon based pn photodiode for near infrared heterostructure photodetector applications. *Appl. Surf. Sci.* **2025**, *687,* 162242.
